# Supplementary material for: Analytical and computational solution for the estimation of SNP-heritability in biobank-scale and distributed datasets
Source: PLoS Comput Biol. 2025 Oct 21;21(10):e1013568. doi: 10.1371/journal.pcbi.1013568 (PMC12539748; doi:10.1371/journal.pcbi.1013568)
Supplement: S1 Text — “Effective number of markers” (Note I); “Discussion of Λ1” (Note II); “Sampling variance for vertical RHE-reg” (Note III); “Adjustment for covariates” (Note IV); “Coding scheme and LD” (Note V). (DOCX) [file pcbi.1013568.s001.docx]

**Title**: Analytical and computational solution for the estimation of SNP-heritability in biobank-scale and distributed datasets

**Authors**: Guo-An Qi^1,2,8^, Qi-Xin Zhang^3,8^, Jingyu Kang^4^, Tianyuan Li^4^, Xiyun Xu^4^, Zhe Zhang^5^, Zhe Fan^6^, Siyang Liu^6^, Guo-Bo Chen^2,7,*^

**Affiliations**:

^1^Institute of Bioinformatics, Zhejiang University, Hangzhou, Zhejiang, China;

^2^Center for Laboratory Medicine, Department of Genetic and Genomic Medicine, and Clinical Research Institute, Zhejiang Provincial People’s Hospital, People’s Hospital of Hangzhou Medical College, Hangzhou, Zhejiang, China;

^3^Department of Epidemiology, School of Public Health, Zhejiang Chinese Medical University, Hangzhou, Zhejiang, China;

^4^School of Mathematics and Statistics and Research Institute of Mathematical Sciences (RIMS), Jiangsu Provincial Key Laboratory of Educational Big Data Science and Engineering, Jiangsu Normal University, Xuzhou, Jiangsu, China;

^5^Department of Animal Science, College of Animal Sciences, Zhejiang University, Hangzhou, Zhejiang, China;

^6^School of Public Health (Shenzhen), Shenzhen Campus of Sun Yat-sen University, Shenzhen, Guangdong, China;

^7^Key Laboratory of Endocrine Gland Diseases of Zhejiang Province, Hangzhou, Zhejiang, China.

^8^**Equal contribution**

**^*^Correspondence**: Guo-Bo Chen (chenguobo@gmail.com)

Table of contents

[Note I: Effective number of markers 3](#_Toc203423923)

[Note II: $\boldsymbol{\Lambda1}$ 4](#_Toc203423924)

[Notes III: Sampling variance for vertical RHE-reg 5](#_Toc203423925)

[Note IV: When there is adjustment for covariates 6](#_Toc203423926)

[Note V: Coding scheme and LD 8](#_Toc203423927)

[Reference 10](#_Toc203423928)

# Note I: Effective number of markers

Estimation for the effective number of markers ($\boldsymbol{m}_{\boldsymbol{e}}$)

$$E\left[ tr\left( \boldsymbol{K}^{2} \right) \right]=E\left\{ \frac{1}{m^{2}}\sum_{i,j}^{n} \left[ \sum_{k}^{m} \left( x_{ik}x_{jk} \right) \right]^{2} \right\}=E\left\{ \frac{1}{m^{2}}\sum_{i,j}^{n} \left\{ \left[ \sum_{k}^{m} \left( x_{ik}x_{jk} \right) \right]\left[ \sum_{l}^{m} \left( x_{il}x_{jl} \right) \right] \right\} \right\}$$

$E\left[ tr\left( \boldsymbol{K}^{2} \right) \right]=\frac{1}{m^{2}}\sum_{i,j}^{n} \left\{ \left[ \sum_{k}^{m} \left( x_{ik}x_{jk} \right) \right]\left[ \sum_{l}^{m} \left( x_{il}x_{jl} \right) \right] \right\}$ can be decomposed into four terms $E\left[ tr\left( \boldsymbol{K}^{2} \right) \right]=\frac{1}{m^{2}}\left[ \sum_{i}^{n} \sum_{k}^{m} x_{ik}^{4}+\sum_{i}^{n} \sum_{k\neq l}^{m} x_{ik}^{2}x_{il}^{2}+\sum_{i\neq j}^{n} \sum_{k}^{m} x_{ik}^{2}x_{jk}^{2}+\sum_{i\neq j}^{n} \sum_{k\neq l}^{m} x_{ik}x_{jk}x_{il}x_{jl} \right]$ upon $i=j$ (or $i\neq j$) and $k=l$ (or $k\neq l$), and according to Isserlis’s Theory [1], we have

1. $\sum_{i}^{n} \sum_{k}^{m} x_{ik}^{4}$: $i=j$ and $k=l$, totaling $nm$ terms. $\sum_{i}^{n} \sum_{k}^{m} x_{ik}^{4}=nm\left[ 3E\left( x_{ik}^{2} \right)E\left( x_{ik}^{2} \right) \right]=3nm$ because $E\left( x_{ik}^{2} \right)E\left( x_{ik}^{2} \right)=1$.
2. $\sum_{i}^{n} \sum_{k\neq l}^{m} x_{ik}^{2}x_{il}^{2}$: $i=j$ and $k\neq l$, totaling $nm(m-1)$ terms. $\sum_{i}^{n} \sum_{k\neq l}^{m} x_{ik}^{2}x_{il}^{2}=n\sum_{k\neq l}^{m} \left( E\left( x_{ik}^{2} \right)E\left( x_{il}^{2} \right)+2E^{2}(x_{ik}x_{il}) \right)=n\sum_{k\neq l}^{m} \left( 1+2\rho_{kl}^{2} \right)$ because $E\left( x_{ik}^{2} \right)E\left( x_{il}^{2} \right)=1$ and $E^{2}\left( x_{ik}x_{il} \right)=\rho_{kl}^{2}$.
3. $\sum_{i\neq j}^{n} \sum_{k}^{m} x_{ik}^{2}x_{jk}^{2}$: $i\neq j$ and $k=l$, totalling $n(n-1)m$ terms. $\sum_{i\neq j}^{n} \sum_{k}^{m} x_{ik}^{2}x_{jk}^{2}=\sum_{i\neq j}^{n} \sum_{k}^{m} [E\left( x_{ik}^{2} \right)E\left( x_{jk}^{2} \right)+2E^{2}\left( x_{ik}x_{jk} \right)]=n\left( n-1 \right)m$ because $E\left( x_{ik}^{2} \right)E\left( x_{jk}^{2} \right)=1$ and $E^{2}\left( x_{ik}x_{jk} \right)=0$.
4. $\sum_{i\neq j}^{n} \sum_{k\neq l}^{m} x_{ik}x_{jk}x_{il}x_{jl}$: $i\neq j$ and $k\neq l$, totaling $n(n-1)m(m-1)$ terms. $\Sigma_{i\neq j}^{n}\sum_{k\neq l}^{m} x_{ik}x_{jk}x_{il}x_{jl}=\Sigma_{i\neq j}^{n}\sum_{k,l}^{m} \left[ E\left( x_{ik}x_{jk} \right)E\left( x_{il}x_{jl} \right)+E\left( x_{ik}x_{il} \right)E\left( x_{jk}x_{jl} \right)+E\left( x_{ik}x_{jl} \right)E\left( x_{jk}x_{il} \right) \right]=n(n-1)\sum_{k\neq l}^{n} \rho_{kl}^{2}$ because $E\left( x_{ik}x_{jk} \right)E\left( x_{il}x_{jl} \right)=0$, $E\left( x_{ik}x_{jl} \right)E\left( x_{jk}x_{il} \right)=0$, and $E\left( x_{ik}x_{il} \right)E\left( x_{jk}x_{jl} \right)=\rho_{kl}^{2}$.

Having integrated these four terms, we have

$$E[tr\left( \boldsymbol{K}^{2} \right)]=\frac{1}{m^{2}}\left[ 3nm+n\sum_{k\neq l}^{m} \left( 1+2\rho_{kl}^{2} \right)+n\left( n-1 \right)m+n\left( n-1 \right)\sum_{k\neq l}^{m} \rho_{kl}^{2} \right]=n\left( n+1 \right)\frac{\sum_{k,l}^{m} \rho_{kl}^{2}}{m^{2}}+n=\frac{n\left( n+1 \right)}{m_{e}}+n$$

in which $m_{e}=\frac{m^{2}}{\sum_{k,l}^{m} \rho_{kl}^{2}}$ the effective number of markers and $\rho_{kl}^{2}$ the squared Pearson’s correlation of LD between a pair of SNPs [2]. Often $m_{e}\leq m$, and $m_{e}=m$ if all markers are in linkage equilibrium (see the note of **Table 1**). Here, $m_{e}$ is a population parameter, a summary statistic that encompasses allelic frequencies and linkage disequilibrium of makers. According to **Eq 3**, $E\left( L_{2,B} \right)=tr\left( \boldsymbol{K}^{2} \right)=\frac{n\left( n+1 \right)}{m_{e}}+n$, we consequently propose a randomization algorithm, which estimates $m_{e}$ as below

| $\left\{ \begin{matrix} \hat{m}_{e}=\frac{n\left( n+1 \right)}{L_{2,B}-n} \\ var\left( \hat{m}_{e} \right)=\frac{{2m}_{e}^{4}}{n^{4}}\frac{tr\left( \boldsymbol{K}^{4} \right)}{B} \end{matrix} \right.$ | (11) |
| --- | --- |

A more detailed estimation procedure for $m_{e}$ can be found in our recent work [3].

# Note II: $\Lambda_{1}$

$$\Lambda_{1}=tr\left\{ \left[ \boldsymbol{\sum}\left( \boldsymbol{K}-\boldsymbol{I}_{n} \right) \right]^{2} \right\}=tr\left\{ \boldsymbol{\sum}\left( \boldsymbol{K}-\boldsymbol{I}_{n} \right)\boldsymbol{\sum}\left( \boldsymbol{K}-\boldsymbol{I}_{n} \right) \right\}=tr\left[ K^{4}h^{4}+2K^{3}\left( h^{2}\sigma_{e}^{2}-h^{4} \right)+2K^{2}\left( h^{2}-\sigma_{e}^{2} \right)^{2}+Kh^{2}\sigma_{e}^{2}+I\sigma_{e}^{4} \right]=tr\left( \boldsymbol{K}^{4} \right)h^{4}+2tr\left( \boldsymbol{K}^{3} \right)\left( h^{2}\sigma_{e}^{2}-h^{4} \right)+2tr\left( \boldsymbol{K}^{2} \right)\left( h^{2}-\sigma_{e}^{2} \right)^{2}+tr\left( \boldsymbol{K} \right)h^{2}\sigma^{2}+tr\left( \boldsymbol{I} \right)\sigma_{e}^{4}\approx tr\left( \boldsymbol{K}^{4} \right)h^{4}+2tr\left( \boldsymbol{K}^{3} \right)h^{2}\left( \sigma_{e}^{2}-h^{2} \right)+2tr\left( \boldsymbol{K}^{2} \right)\left( h^{2}-\sigma_{e}^{2} \right)^{2}+n\sigma_{e}^{2}(h^{2}+\sigma_{e}^{2})\approx tr\left( \boldsymbol{K}^{4} \right)h^{4}+2tr\left( \boldsymbol{K}^{3} \right)h^{2}\left( \sigma_{e}^{2}-h^{2} \right)+2tr\left( \boldsymbol{K}^{2} \right)\left( h^{2}-\sigma_{e}^{2} \right)^{2}+n\sigma_{e}^{2}$$

It exists three methods to compute $\Lambda_{1}$.

Method 1

In our paper, we express it as by replacing $\Sigma=Kh^{2}+I\sigma_{e}^{2}$ with $\boldsymbol{y}\boldsymbol{y}^{T}$

| $\Lambda_{1}\approx\left\{ \boldsymbol{y}^{T}\left( \boldsymbol{K}-\boldsymbol{I} \right)\boldsymbol{K}\left( \boldsymbol{K}-\boldsymbol{I} \right)\boldsymbol{y}\hat{h}^{2}+\boldsymbol{y}^{T}\left( \boldsymbol{K}-\boldsymbol{I} \right)\left( \boldsymbol{K}-\boldsymbol{I} \right)\boldsymbol{y}\hat{\sigma}_{e}^{2} \right\}\approx\left[ L_{3,0}-2L_{2,0}+L_{1,0} \right]\hat{h}^{2}+\left[ L_{2,0}-2L_{1,0}+n \right]\hat{\sigma}_{e}^{2}=\mathcal{L}_{h^{2}}\hat{h}^{2}+\mathcal{L}_{\sigma_{e}^{2}}\hat{\sigma}_{e}^{2}$ | (12) |
| --- | --- |

Method 2

If sample size is large, an randomized estimation for $\Lambda_{1}$ if we replace $tr\left( \boldsymbol{K}^{c} \right)$ with $L_{c,B}=\frac{1}{B}\sum_{b=1}^{B} \boldsymbol{z}_{b}^{T}K^{c}\boldsymbol{z}_{b}$ [4,5].

Method 3

For small data, as $tr\left( \boldsymbol{K}^{c} \right)=\sum_{j=1}^{K} \lambda_{j}^{c}$, for small data we can directly estimate its eigenvalues of $\boldsymbol{K}$.

# Notes III: Sampling variance for vertical RHE-reg

For vertical RHE-reg

$${\hat{\tilde{h}}}^{2}=\frac{\tilde{n}^{2}(\boldsymbol{y}^{T}\boldsymbol{Ky}-n)}{n^{2}(\tilde{L}_{2,B}-\tilde{n})}$$

If the numerator and denominator are estimated from different datasets, according to Delta method, its sampling variance can be expressed as $var\left( \frac{a}{b} \right)=\frac{1}{\mu_{b}^{2}}\sigma_{a}^{2}-2\frac{\mu_{a}}{\mu_{b}^{3}}cov\left( a,b \right)+\frac{\mu_{a}^{2}}{\mu_{b}^{4}}\sigma_{b}^{2}$, in which the covariance term can be zeroed out in this scenario,

$$\left\{ \begin{aligned} a\left\{ \begin{matrix} \mu_{a}=E\left( y^{T}Ky-n \right)=\left[ tr\left( \boldsymbol{K}^{2} \right)-n \right]h^{2} \\ \sigma_{a}^{2}=var\left( \boldsymbol{y}^{T}\left( \boldsymbol{K}-I \right)\boldsymbol{y} \right)=2tr[\Sigma(K-I)\Sigma(K-I)] \end{matrix} \right. \\ b\left\{ \begin{matrix} \mu_{b}=\tilde{L}_{2,B}-\tilde{n}=tr\left( \tilde{K}^{2} \right)-\tilde{n}=\frac{\tilde{n}(\tilde{n}+1)}{\tilde{m}_{e}} \\ \sigma_{b}^{2}=\frac{2}{B}tr(\tilde{K}^{4}) \end{matrix} \right. \end{aligned} \right.$$

$$var\left( \frac{a}{b} \right)=\frac{1}{\mu_{b}^{2}}\sigma_{a}^{2}+\frac{\mu_{a}^{2}}{\mu_{b}^{4}}\sigma_{b}^{2}=\frac{1}{tr\left( \tilde{K}^{2} \right)-\tilde{n}}2tr\left[ \Sigma\left( K-I \right)\Sigma\left( K-I \right) \right]+\frac{\left\{ \left[ tr\left( \boldsymbol{K}^{2} \right)-n \right]h^{2} \right\}^{2}}{\left[ tr\left( \tilde{K}^{2} \right)-\tilde{n} \right]^{4}}\frac{2}{B}tr\left( \tilde{K}^{4} \right)=\frac{1}{\left[ tr\left( \tilde{K}^{2} \right)-\tilde{n} \right]^{2}}[2tr\left[ \Sigma\left( K-I \right)\Sigma\left( K-I \right) \right]+\left[ \frac{tr\left( \boldsymbol{K}^{2} \right)-n}{tr\left( {\tilde{\boldsymbol{K}}}^{2} \right)-\tilde{n}} \right]^{2}h^{4}\frac{2}{B}tr\left( \tilde{K}^{4} \right)]$$

As $E\left[ tr\left( K^{2} \right) \right]=\frac{n^{2}}{m_{e}}+n$, and $E\left[ tr\left( \tilde{K}^{2} \right) \right]=\frac{\tilde{n}^{2}}{\tilde{m}_{e}}+\tilde{n}$. So $\left[ \frac{tr\left( \boldsymbol{K}^{2} \right)-n}{tr\left( {\tilde{\boldsymbol{K}}}^{2} \right)-\tilde{n}} \right]^{2}=\left( \frac{n^{2}}{\tilde{n}^{2}}\frac{\tilde{m}_{e}}{m_{e}} \right)^{2}$

$$var\left( \frac{a}{b} \right)\approx\left( \frac{\tilde{n}^{2}}{n^{2}} \right)^{2}\left( \frac{\tilde{m}_{e}}{\tilde{n}^{2}} \right)^{2}\left[ \Lambda_{1}+\left( \frac{n^{2}}{\tilde{n}^{2}}\frac{\tilde{m}_{e}}{m_{e}} \right)^{2}h^{4}\frac{2}{B}tr\left( \tilde{K}^{4} \right) \right]$$

If the reference panel is of similar sample size to

$$var\left( \frac{a}{b} \right)\approx\left( \frac{\tilde{m}_{e}}{\tilde{n}^{2}} \right)^{2}\left[ \Lambda_{1}+\left( \frac{\tilde{m}_{e}}{m_{e}} \right)^{2}h^{4}\frac{2}{B}tr\left( \tilde{K}^{4} \right) \right]$$

If furthermore, the numerator and denominator are from the same ancestry

$$var\left( \frac{a}{b} \right)\approx\left( \frac{\tilde{m}_{e}}{\tilde{n}^{2}} \right)^{2}\left[ \Lambda_{1}+h^{4}\frac{2}{B}tr\left( \tilde{K}^{4} \right) \right]$$

# Note IV: When there is adjustment for covariates

We can derive the equation below with the inclusion of covariates.

$$\left[ \begin{matrix} tr\left( \boldsymbol{KVKV} \right) & tr\left( \boldsymbol{KV} \right) \\ tr\left( \boldsymbol{KV} \right) & tr\left( \boldsymbol{V} \right) \end{matrix} \right]\left[ \begin{matrix} \tilde{h}^{2} \\ \tilde{\sigma}^{2} \end{matrix} \right]=\left[ \begin{matrix} {\tilde{\boldsymbol{y}}}^{T}\boldsymbol{K}\tilde{\boldsymbol{y}} \\ {\tilde{\boldsymbol{y}}}^{T}\tilde{\boldsymbol{y}} \end{matrix} \right]$$

In which $\boldsymbol{V}=\boldsymbol{I}-\boldsymbol{W}\left( \boldsymbol{W}^{T}\boldsymbol{W} \right)^{-1}\boldsymbol{W}^{T}$ and the covariance matrix $\boldsymbol{W}$ is $n\times w$ matrix, MSE becomes

| $\left[ \begin{matrix} \tilde{h}^{2} \\ \tilde{\sigma}^{2} \end{matrix} \right]=\left\{ \begin{matrix} \frac{{\tilde{\boldsymbol{y}}}^{T}[tr\left( \boldsymbol{V} \right)\boldsymbol{K}-tr\left( \boldsymbol{KV} \right)\boldsymbol{I}]\tilde{\boldsymbol{y}}}{\left( L_{B}+\mathcal{c} \right)tr\left( \boldsymbol{V} \right)-tr^{2}\left( \boldsymbol{KV} \right)} \\ \frac{{\tilde{\boldsymbol{y}}}^{T}[-tr\left( \boldsymbol{KV} \right)\boldsymbol{K}+\left( L_{B}+\mathcal{c} \right)]\tilde{\boldsymbol{y}}}{tr\left( \boldsymbol{V} \right)(L_{B}+\mathcal{c})-tr^{2}\left( \boldsymbol{KV} \right)} \end{matrix} \right.$ | (A1) |
| --- | --- |

| $MSE\left( {\hat{\tilde{h}}}^{2} \right)=\frac{2tr\{\left\{ \tilde{\Sigma}\left[ tr\left( \boldsymbol{V} \right)\boldsymbol{K}-tr\left( \boldsymbol{KV} \right)\boldsymbol{I} \right] \right\}^{2}\}}{\left[ tr\left( \boldsymbol{V} \right)tr\left( \boldsymbol{KVKV} \right)-tr^{2}\left( \boldsymbol{KV} \right) \right]^{2}}+\frac{1}{B}\frac{tr^{2}\left( \boldsymbol{V} \right)\cdot2tr(\boldsymbol{K}^{4})}{\left[ tr\left( \boldsymbol{V} \right)tr\left( \boldsymbol{KVKV} \right)-tr^{2}\left( \boldsymbol{KV} \right) \right]^{2}}{\hat{\tilde{h}}}_{g}^{4}+\frac{1}{B^{2}}\left[ \frac{tr^{2}\left( \boldsymbol{V} \right)\cdot2tr\left( \boldsymbol{K}^{4} \right)}{\left[ tr\left( \boldsymbol{V} \right)tr\left( \boldsymbol{KVKV} \right)-tr^{2}\left( \boldsymbol{KV} \right) \right]^{2}} \right]^{2}{\hat{\tilde{h}}}_{g}^{4}$ | (A2) |
| --- | --- |

in which $\boldsymbol{V}=\boldsymbol{I}_{n}-\boldsymbol{W}\left( \boldsymbol{W}^{T}\boldsymbol{W} \right)^{-1}\boldsymbol{W}^{T}$. And consequently,

| $B\geq\frac{tr^{2}\left( \boldsymbol{V} \right)\cdot2tr(\boldsymbol{K}^{4})\hat{h}^{4}}{\eta tr\{\left\{ \tilde{\boldsymbol{\Sigma}}\left[ tr\left( \boldsymbol{V} \right)\boldsymbol{K}-tr\left( \boldsymbol{KV} \right)\boldsymbol{I}_{n} \right] \right\}^{2}\}}$ | (A3) |
| --- | --- |

When there is covariance matrix $\boldsymbol{W}$, which is $n\times w$ matrix, MSE becomes

| $MSE\left( \hat{h}^{2} \right)=\frac{2tr\{\left\{ \tilde{\Sigma}\left[ tr\left( \boldsymbol{V} \right)\boldsymbol{K}-tr\left( \boldsymbol{KV} \right)\boldsymbol{I} \right] \right\}^{2}\}}{\left[ tr\left( \boldsymbol{K}^{\boldsymbol{2}} \right)-n \right]^{2}}+\frac{1}{B}\frac{\left[ tr\left( \boldsymbol{V} \right)tr\left( \boldsymbol{KVKV} \right)-tr^{2}\left( \boldsymbol{KV} \right) \right]^{2}\cdot tr^{2}\left( \boldsymbol{V} \right)\cdot2tr(\boldsymbol{K}^{4})}{\left[ tr\left( \boldsymbol{K}^{\boldsymbol{2}} \right)-n \right]^{4}}h_{g}^{4}+\frac{1}{B^{2}}\left[ \frac{tr^{2}\left( \boldsymbol{V} \right)\cdot2tr\left( \boldsymbol{K}^{4} \right)}{\left[ tr\left( \boldsymbol{K}^{\boldsymbol{2}} \right)-n \right]^{2}} \right]^{2}h_{g}^{4}$ | (A4) |
| --- | --- |

in which $\boldsymbol{V}=\boldsymbol{I}_{n}-\boldsymbol{W}\left( \boldsymbol{W}^{T}\boldsymbol{W} \right)^{-1}\boldsymbol{W}^{T}$. And consequently,

| $B\geq\frac{\left[ tr\left( \boldsymbol{V} \right)tr\left( \boldsymbol{KVKV} \right)-tr^{2}(\boldsymbol{KV}) \right]^{2}\cdot tr^{2}\left( \boldsymbol{V} \right)\cdot2tr(\boldsymbol{K}^{4})\hat{h}^{4}}{\eta\left[ tr\left( \boldsymbol{K}^{2} \right)-n \right]^{2}\cdot tr\{\left\{ \tilde{\boldsymbol{\Sigma}}\left[ tr\left( \boldsymbol{V} \right)\boldsymbol{K}-tr\left( \boldsymbol{KV} \right)\boldsymbol{I}_{n} \right] \right\}^{2}\}}$ | (A5) |
| --- | --- |

And a hybrid one

$$\left[ \begin{matrix} tr\left( \boldsymbol{K}^{\boldsymbol{2}} \right) & tr\left( \boldsymbol{K} \right) \\ tr\left( \boldsymbol{K} \right) & n \end{matrix} \right]\left[ \begin{matrix} \breve{h}^{2} \\ \breve{\sigma}^{2} \end{matrix} \right]=\left[ \begin{matrix} {\tilde{\boldsymbol{y}}}^{T}\boldsymbol{K}\tilde{\boldsymbol{y}} \\ {\tilde{\boldsymbol{y}}}^{T}\tilde{\boldsymbol{y}} \end{matrix} \right]$$

| $\left[ \begin{matrix} {\hat{\breve{h}}}^{2} \\ {\hat{\breve{\sigma}}}^{2} \end{matrix} \right]=\left\{ \begin{aligned} \frac{{\tilde{\boldsymbol{y}}}^{T}(n\boldsymbol{K}-tr(\boldsymbol{K})\boldsymbol{I}_{n})\tilde{\boldsymbol{y}}}{n[tr(\boldsymbol{K}^{2})-n]} \\ \frac{{\tilde{\boldsymbol{y}}}^{T}[tr\left( \boldsymbol{K}^{2} \right)\boldsymbol{I}_{n}-\boldsymbol{K}tr\left( \boldsymbol{K} \right)\boldsymbol{]}\tilde{\boldsymbol{y}}}{ntr(\boldsymbol{K}^{2})-n^{2}} \end{aligned} \right.$ | (A6) |
| --- | --- |

| $var ({\hat{\breve{h}}}^{2})=\frac{2tr\left\{ \left[ \breve{\boldsymbol{\sum}}\left( \boldsymbol{K}-\boldsymbol{I}_{n} \right) \right]^{2} \right\}}{[tr(\boldsymbol{K}^{2})-n]^{2}}+\frac{2}{B}\frac{tr(\boldsymbol{K}^{4})}{\left[ tr\left( \boldsymbol{K}^{2} \right)-n \right]^{2}}{\hat{\breve{h}}}^{4}$ | (A7) |
| --- | --- |

$$tr\left\{ \left[ \breve{\boldsymbol{\sum}}\left( \boldsymbol{K}-\boldsymbol{I}_{n} \right) \right]^{2} \right\}\approx\left\{ {\tilde{\boldsymbol{y}}}^{T}\left( \boldsymbol{K}-\boldsymbol{I} \right)\boldsymbol{K}\left( \boldsymbol{K}-\boldsymbol{I} \right)\tilde{\boldsymbol{y}}{\hat{\breve{h}}}^{2}+{\tilde{\boldsymbol{y}}}^{T}\left( \boldsymbol{K}-\boldsymbol{I} \right)\left( \boldsymbol{K}-\boldsymbol{I} \right)\tilde{\boldsymbol{y}}{\hat{\breve{\sigma}}}^{2} \right\}\approx\left[ L_{3,\tilde{y}}-2L_{2,\tilde{y}}+L_{1,\tilde{y}} \right]{\hat{\breve{h}}}^{2}+\left[ L_{2,\tilde{y}}-2L_{1,\tilde{y}}+n \right]{\hat{\breve{\sigma}}}^{2}$$

At the same time, we can also find the mean squared error (*MSE*) for $\hat{h}^{2}$ as below

| $MSE\left( {\hat{\breve{h}}}^{2} \right)=var\left( {\hat{\breve{h}}}^{2} \right)+\Delta^{2}=\frac{2tr\left\{ \left[ \breve{\boldsymbol{\sum}}\left( \boldsymbol{K}-\boldsymbol{I}_{n} \right) \right]^{2} \right\}}{[tr(\boldsymbol{K}^{2})-n]^{2}}+\frac{2}{B}\frac{tr(\boldsymbol{K}^{4})}{\left[ tr\left( \boldsymbol{K}^{2} \right)-n \right]^{2}}{\hat{\breve{h}}}^{4}+\left\{ \frac{2}{B}\frac{tr\left( \boldsymbol{K}^{4} \right)}{[tr(\boldsymbol{K}^{2})-n]^{2}} \right\}^{2}{\hat{\breve{h}}}^{4}$ | (A8) |
| --- | --- |

$$B\geq\frac{tr\left( \boldsymbol{K}^{4} \right){\hat{\breve{h}}}^{4}}{\eta tr\left\{ \left[ \breve{\boldsymbol{\Sigma}}\left( \boldsymbol{K}-\boldsymbol{I}_{n} \right) \right]^{2} \right\}}$$

# Note V: Coding scheme and LD

Coding table for a pair of loci

| **Genotype** $\boldsymbol{x}_{\boldsymbol{i,k}}\boldsymbol{x}_{\boldsymbol{i,l}}$ | **Coding scheme^1,2^** | **Frequencies for** $\boldsymbol{x}_{\boldsymbol{i,k}}\boldsymbol{x}_{\boldsymbol{i,l}}$ **(**$\boldsymbol{f}_{\boldsymbol{v}_{\boldsymbol{1}}\boldsymbol{v}_{\boldsymbol{2}}}$**)** |
| --- | --- | --- |
| $A_{k}A_{k}B_{l}B_{l}$ | $\alpha_{1}\beta_{1}$ | $f_{1,1}=p_{k}^{2}R_{kl}^{2}=p_{k}^{2}p_{l}^{2}+2p_{k}p_{l}D_{kl}+D_{kl}^{2}$ |
| $A_{k}A_{k}B_{l}b_{l}$ | $\alpha_{1}\beta_{2}$ | $f_{1,2}=p_{k}^{2}\cdot2R_{kl}\bar{R}_{kl}=2p_{k}^{2}p_{l}q_{l}+2p_{k}\left( p_{l}-q_{l} \right)D_{kl}-2D_{kl}^{2}$ |
| $A_{k}A_{k}b_{l}b_{l}$ | $\alpha_{1}\beta_{3}$ | $f_{1,3}=p_{k}^{2}\bar{R}_{kl}^{2}=p_{k}^{2}q_{l}^{2}-2p_{k}q_{l}D_{kl}+D_{kl}^{2}$ |
| $A_{k}a_{k}B_{l}B_{l}$ | $\alpha_{2}\beta_{1}$ | $f_{2,1}=2p_{k}q_{k}R_{kl}\bar{r}_{kl}=2p_{k}q_{k}p_{l}^{2}+2p_{l}\left( p_{k}-q_{k} \right)D_{kl}-2D_{kl}^{2}$ |
| $A_{k}a_{k}B_{l}b_{l}$ | $\alpha_{2}\beta_{2}$ | $f_{2,2}=2p_{k}q_{k}\left( \bar{R}_{kl}\bar{r}_{kl}+R_{kl}r_{kl} \right)=4p_{k}q_{k}p_{l}q_{l}+2\left( p_{k}-q_{k} \right)\left( p_{l}-q_{l} \right)D_{kl}+4D_{kl}^{2}$ |
| $A_{k}a_{k}b_{l}b_{l}$ | $\alpha_{2}\beta_{3}$ | $f_{2,3}=2p_{k}q_{k}\bar{R}_{kl}r_{kl}=2p_{k}q_{k}q_{l}^{2}+2q_{l}\left( p_{k}-q_{k} \right)D_{kl}-2D_{kl}^{2}$ |
| $a_{k}a_{k}B_{l}B_{l}$ | $\alpha_{3}\beta_{1}$ | $f_{3,1}=q_{k}^{2}\bar{r}_{kl}^{2}=q_{k}^{2}p_{l}^{2}-2q_{k}p_{l}D_{kl}+D_{kl}^{2}$ |
| $a_{k}a_{k}B_{l}b_{l}$ | $\alpha_{3}\beta_{2}$ | $f_{3,2}=2p_{k}q_{k}\bar{r}_{kl}r_{kl}=2q_{k}^{2}p_{l}q_{l}+2q_{k}\left( p_{l}-q_{l} \right)D_{kl}-2D_{kl}^{2}$ |
| $a_{k}a_{k}b_{l}b_{l}$ | $\alpha_{3}\beta_{3}$ | $f_{3,3}=q_{k}^{2}r_{kl}^{2}=q_{k}^{2}q_{l}^{2}+2q_{k}q_{l}D_{kl}+D_{kl}^{2}$ |

^1^For additive effect, under the coding scheme of 0 ($aa$), 1 ($Aa$), and 2 ($AA$) that counts the number of reference allele ($A$), which has allele frequency of $p$; $q=1-p$ is the frequency of the alternative allele. After standardizing each genotype, we have $\left[ \alpha_{1},\alpha_{2},\alpha_{3} \right]=[\frac{2q_{k}}{\sqrt{2p_{k}q_{k}}},\frac{q_{k}-p_{k}}{\sqrt{2p_{k}q_{k}}},\frac{-2p_{k}}{\sqrt{2p_{k}q_{k}}}]$ for $AA$, $Aa$, and $aa$, and $\left[ \beta_{1},\beta_{2},\beta_{3} \right]=[\frac{2q_{l}}{\sqrt{2p_{l}q_{l}}},\frac{q_{l}-p_{l}}{\sqrt{2p_{l}q_{l}}},\frac{-2p_{l}}{\sqrt{2p_{l}q_{l}}}]$ for $BB$, $Bb$, and $bb$.

It leads to

$\sum_{v_{1},v_{2}}^{3} f_{v_{1}v_{2}}\alpha_{v_{1}}\beta_{v_{2}}=\left( p_{k}^{2}p_{l}^{2}+2p_{k}p_{l}D_{kl}+D_{kl}^{2} \right)\frac{2q_{k}}{\sqrt{2p_{k}q_{k}}}\frac{2q_{l}}{\sqrt{2p_{l}q_{l}}}+\left( 2p_{k}^{2}p_{l}q_{l}+2p_{k}\left( p_{l}-q_{l} \right)D_{kl}-2D_{kl}^{2} \right)\frac{2q_{k}}{\sqrt{2p_{k}q_{k}}}\frac{q_{l}-p_{l}}{\sqrt{2p_{l}q_{l}}}+\left( p_{k}^{2}q_{l}^{2}-2p_{k}q_{l}D_{kl}+D_{kl}^{2} \right)\frac{2q_{k}}{\sqrt{2p_{k}q_{k}}}\frac{-2p_{l}}{\sqrt{2p_{l}q_{l}}}+\left( 2p_{k}q_{k}p_{l}^{2}+2p_{l}\left( p_{k}-q_{k} \right)D_{kl}-2D_{kl}^{2} \right)\frac{q_{k}-p_{k}}{\sqrt{2p_{k}q_{k}}}\frac{2q_{l}}{\sqrt{2p_{l}q_{l}}}+\left( 4p_{k}q_{k}p_{l}q_{l}+2\left( p_{k}-q_{k} \right)\left( p_{l}-q_{l} \right)D_{kl}+4D_{kl}^{2} \right)\frac{q_{k}-p_{k}}{\sqrt{2p_{k}q_{k}}}\frac{q_{l}-p_{l}}{\sqrt{2p_{l}q_{l}}}+\left( 2p_{k}q_{k}q_{l}^{2}+2q_{l}\left( p_{k}-q_{k} \right)D_{kl}-2D_{kl}^{2} \right)\frac{q_{k}-p_{k}}{\sqrt{2p_{k}q_{k}}}\frac{-2p_{l}}{\sqrt{2p_{l}q_{l}}}+\left( q_{k}^{2}p_{l}^{2}-2q_{k}p_{l}D_{kl}+D_{kl}^{2} \right)\frac{-2p_{k}}{\sqrt{2p_{k}q_{k}}}\frac{2q_{l}}{\sqrt{2p_{l}q_{l}}}+\left( 2q_{k}^{2}p_{l}q_{l}+2q_{k}\left( p_{l}-q_{l} \right)D_{kl}-2D_{kl}^{2} \right)\frac{-2p_{k}}{\sqrt{2p_{k}q_{k}}}\frac{q_{l}-p_{l}}{\sqrt{2p_{l}q_{l}}}+\left( q_{k}^{2}q_{l}^{2}+2q_{k}q_{l}D_{kl}+D_{kl}^{2} \right)\frac{-2p_{k}}{\sqrt{2p_{k}q_{k}}}\frac{-2p_{l}}{\sqrt{2p_{l}q_{l}}}=\frac{D_{kl}}{\sqrt{2p_{k}q_{k}2p_{l}q_{l}}}=\rho_{kl}$.

For GRM based on additive coding, for individual $i$ and $j$, $\left( \sum_{v_{1},v_{2}}^{3} f_{v_{1}v_{2}}\alpha_{v_{1}}\beta_{v_{2}} \right)_{i}\left( \sum_{v_{1},v_{2}}^{3} f_{v_{1}v_{2}}\alpha_{v_{1}}\beta_{v_{2}} \right)_{j}$ it is easy to see the relationship will be about $\rho_{kl}^{2}$.

^2^For dominance effect, under the coding scheme of 0 ($aa$), $2p_{l}$ ($Aa$), and $4p_{l}-2$ ($AA$) for 0, 1, and 2 reference alleles, we have $\left[ \alpha_{1},\alpha_{2},\alpha_{3} \right]=[\frac{-2q_{k}^{2}}{\sqrt{4p_{k}^{2}q_{k}^{2}}},\frac{2p_{k}q_{k}}{\sqrt{4p_{k}^{2}q_{k}^{2}}},\frac{-2p_{k}^{2}}{\sqrt{4p_{k}^{2}q_{k}^{2}}},]$ for $AA$, $Aa$, and $aa$, and $\left[ \beta_{1},\beta_{2},\beta_{3} \right]=[\frac{-2q_{l}^{2}}{\sqrt{4p_{l}^{2}q_{l}^{2}}},\frac{2p_{l}q_{l}}{\sqrt{4p_{l}^{2}q_{l}^{2}}},\frac{-2p_{l}^{2}}{\sqrt{4p_{l}^{2}q_{l}^{2}}}]$ for $BB$, $Bb$, and $bb$. It leads to

$$\sum_{v_{1},v_{2}}^{3} f_{v_{1}v_{2}}\alpha_{v_{1}}\beta_{v_{2}}=\frac{4D_{kl}^{2}}{\sqrt{4p_{k}^{2}q_{k}^{2}\cdot4p_{l}^{2}q_{l}^{2}}}=\left( p_{k}^{2}p_{l}^{2}+2p_{k}p_{l}D_{kl}+D_{kl}^{2} \right)\frac{-2q_{k}^{2}}{\sqrt{4p_{k}^{2}q_{k}^{2}}}\frac{-2q_{l}^{2}}{\sqrt{4p_{l}^{2}q_{l}^{2}}}+\left( 2p_{k}^{2}p_{l}q_{l}+2p_{k}\left( p_{l}-q_{l} \right)D_{kl}-2D_{kl}^{2} \right)\frac{-2q_{k}^{2}}{\sqrt{4p_{k}^{2}q_{k}^{2}}}\frac{2p_{l}q_{l}}{\sqrt{4p_{l}^{2}q_{l}^{2}}}+\left( p_{k}^{2}q_{l}^{2}-2p_{k}q_{l}D_{kl}+D_{kl}^{2} \right)\frac{-2q_{k}^{2}}{\sqrt{4p_{k}^{2}q_{k}^{2}}}\frac{-2p_{l}^{2}}{\sqrt{4p_{l}^{2}q_{l}^{2}}}+\left( 2p_{k}q_{k}p_{l}^{2}+2p_{l}\left( p_{k}-q_{k} \right)D_{kl}-2D_{kl}^{2} \right)\frac{2p_{k}q_{k}}{\sqrt{4p_{k}^{2}q_{k}^{2}}}\frac{-2q_{l}^{2}}{\sqrt{4p_{l}^{2}q_{l}^{2}}}+\left( 4p_{k}q_{k}p_{l}q_{l}+2\left( p_{k}-q_{k} \right)\left( p_{l}-q_{l} \right)D_{kl}+4D_{kl}^{2} \right)\frac{2p_{k}q_{k}}{\sqrt{4p_{k}^{2}q_{k}^{2}}}\frac{2p_{l}q_{l}}{\sqrt{4p_{l}^{2}q_{l}^{2}}}+\left( 2p_{k}q_{k}q_{l}^{2}+2q_{l}\left( p_{k}-q_{k} \right)D_{kl}-2D_{kl}^{2} \right)\frac{2p_{k}q_{k}}{\sqrt{4p_{k}^{2}q_{k}^{2}}}\frac{-2p_{l}^{2}}{\sqrt{4p_{l}^{2}q_{l}^{2}}}+\left( q_{k}^{2}p_{l}^{2}-2q_{k}p_{l}D_{kl}+D_{kl}^{2} \right)\frac{-2p_{k}^{2}}{\sqrt{4p_{k}^{2}q_{k}^{2}}}\frac{-2q_{l}^{2}}{\sqrt{4p_{l}^{2}q_{l}^{2}}}+\left( 2q_{k}^{2}p_{l}q_{l}+2q_{k}\left( p_{l}-q_{l} \right)D_{kl}-2D_{kl}^{2} \right)\frac{-2p_{k}^{2}}{\sqrt{4p_{k}^{2}q_{k}^{2}}}\frac{2p_{l}q_{l}}{\sqrt{4p_{l}^{2}q_{l}^{2}}}+\left( q_{k}^{2}q_{l}^{2}+2q_{k}q_{l}D_{kl}+D_{kl}^{2} \right)\frac{-2p_{k}^{2}}{\sqrt{4p_{k}^{2}q_{k}^{2}}}\frac{-2p_{l}^{2}}{\sqrt{4p_{l}^{2}q_{l}^{2}}}=\rho_{kl}^{2}$$

For GRM based on additive coding, for individual $i$ and $j$, $\left( \sum_{v_{1},v_{2}}^{3} f_{v_{1}v_{2}}\alpha_{v_{1}}\beta_{v_{2}} \right)_{i}\left( \sum_{v_{1},v_{2}}^{3} f_{v_{1}v_{2}}\alpha_{v_{1}}\beta_{v_{2}} \right)_{j}$ it is easy to see the relationship will be about $\rho_{kl}^{4}$.

# Reference

1. Isserlis L. On a formula for the product-moment coefficient of any order of a normal frequency distribution in any number of variables. Biometrika. 1918;12: 134–139. doi:10.2307/2331932

2. Goddard M. Genomic selection: prediction of accuracy and maximisation of long term response. Genetica. 2009;136: 245–257. doi:10.1007/s10709-008-9308-0

3. Zhang Q-X, Jayasinghe D, Zhang Z, Lee SH, Xu H, Chen G. Precise estimation of in-depth relatedness in biobank-scale datasets using deepKin. Cell Reports Methods. 2025;5: 101053. doi:10.1016/j.crmeth.2025.101053

4. Girard DA. A Fast “Monte-Carlo Cross-Validation” procedure for Large Least Squares Problems with Noisy Data. Numer Math. 1989;56: 1–24.

5. Hutchinson MF. A stochastic estimator of the trace of the influence matrix for laplacian smoothing splines. Commun Stat - Simul Comput. 1989;18: 1059–1076. doi:10.1080/03610918908812806
