# Supplementary figures and images for: Analytical and computational solution for the estimation of SNP-heritability in biobank-scale and distributed datasets
Source: PLoS Comput Biol. 2025 Oct 21;21(10):e1013568. doi: 10.1371/journal.pcbi.1013568 (PMC12539748; doi:10.1371/journal.pcbi.1013568)

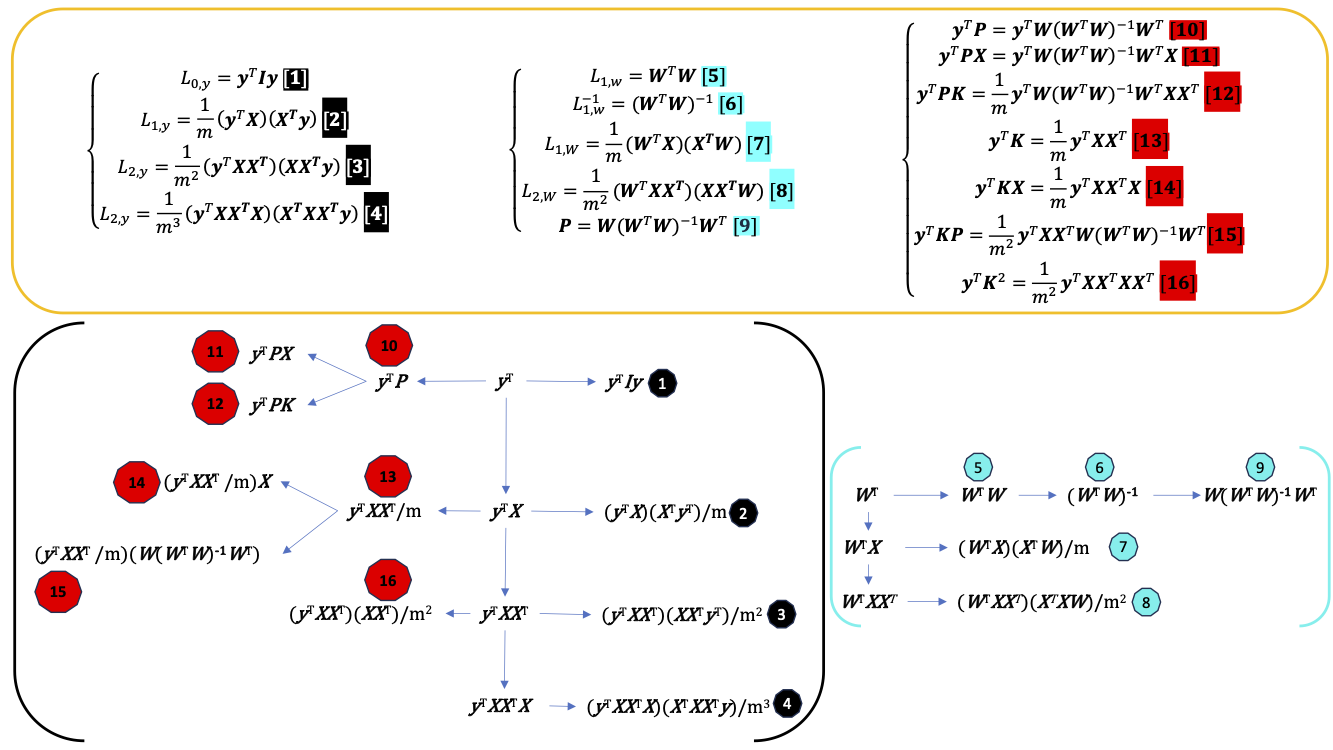

Supplement: S1 Fig — Some sequential operation of the matrix is suggested to make the program easy to write. (TIFF) [file pcbi.1013568.s002.tiff]
