# Supplementary material for: Analytical and computational solution for the estimation of SNP-heritability in biobank-scale and distributed datasets
Source: PLoS Comput Biol. 2025 Oct 21;21(10):e1013568. doi: 10.1371/journal.pcbi.1013568 (PMC12539748; doi:10.1371/journal.pcbi.1013568)
Supplement: Attachment — Submitted filename: PLoSCompBiol_Review.Jul13.cgb.docx [file pcbi.1013568.s007.docx]

**Reviewer #1**

**In the manuscript “Analytical and computational solution for the estimation of SNP-heritability in biobank- scale and distributed datasets” by Qi et al., the authors describe an improved implementation of the randomized Haseman-Elston regression (RHE-reg) to estimate SNP heritability on biobank-scale data (i.e., genomic analysis with hundreds of thousands of samples genotyped with hundreds of thousands SNP). The methodology is extended to distributed data (i.e., analysis on genomic data coming from different sources under privacy). The work presents an analytical procedure to control the number of iterations of the RHE-reg, which has been shown to be a limitation of the RHE-reg. A free software is mentioned (**[**https://github.com/gc5k/gear2**](https://github.com/gc5k/gear2)**), albeit without further information on the software. The work is based on previous works on the RHE-reg trying to address limitation on the number of iterations needed to provide with accurate solutions. Moreover, given the plethora of available software that can handle biobank-scale genomic data, the novelty of the work is questionable. Overall, the topic is of interest to the readership of PLOS Computational Biology in the field of genetics/genomics. The manuscript is well written. Despite this, there are some critical points that need to be addressed:**

Reply: Thanks for your appreciation.

**Major comments**

**1 The authors stated that the aim of the work is to estimate SNP-heritability for biobank-scale data. However, in the simulation the number of samples was set to 1,000, 5,000, and 10,000, and the number of SNPs to 10,000, 50,000, and 100,000. None of this combinations is close to what is known as biobank-scale. Moreover, more details should be given for the simulation scenario, e.g., software used and relationship among samples (related or unrelated individuals were simulated?).**

Reply: Unrelated individuals were simulated in this study to test the statistical properties of the proposed method, and the rationale of the method is from GWAS for complex traits in a recent decade or even longer using unrelated individuals. So, in general, the theory and practice of estimating unrelated individuals for heritability has been a while, and the current bottleneck is to conquer biobank-scale data. The study that you are reviewing now represents such an attempt. The direct source of the study is from these three papers below:

Guo-Bo Chen, Estimating heritability of complex traits from genome-wide association studies using IBS-based Haseman-Elston regression, ***Front Genet***, 2014, 5:107; this paper gives a method-of-moment framework for estimating heritability for complex traits and provides analytical solution for the estimated heritability. However, the involvement of $\boldsymbol{K}=\frac{1}{m}\boldsymbol{X}\boldsymbol{X}^{T}$ leads to high computational cost and not a good tool for biobank-scale data (from the author himself).

Xiang Zhou, A unified framework for variance component estimation with summary statistics in genome-wide association studies, ***Annals of Applied Statistics***, 2017, 11:2027-51; this paper gives the identity of $tr\left( \boldsymbol{K}^{2} \right)=\frac{n^{2}}{m_{e}}+n$, but it fails to estimate $m_{e}$ in biobank scale datasets.

Yue Wu, Sriram Sankararaman, A scalable estimator of SNP heritability for biobank-scale data, ***Bioinformatics***, 2018, 34:i187-194. This paper smartly introduces Girard-Hutchison estimation to solve $tr(\boldsymbol{K}^{2})$, but made mistakes and eventually lost control of how many iteration should be if a desired precision is needed.

In terms of the simulated data, because we need to validate some ground truth. For example, $\boldsymbol{K}=\frac{1}{m}\boldsymbol{X}\boldsymbol{X}^{T}$, which is computationally expensive, but some benchmark properties of $\boldsymbol{K}$ such as $tr\left( \boldsymbol{K}^{4} \right)$ should be validated with classical methods, which need to calculate $\sum_{i=1}^{n} \lambda_{i}^{4}$. $\lambda_{i}$ the $i^{th}$ eigenvalue of $\boldsymbol{K}$. So, only limited sample size and SNP numbers were simulated, otherwise would be very time-consuming to validate the ground truth.

**For your inquiry “None of this combinations is close to what is known as biobank-scale.”**

For example, we increased $n$ to 20,000 and $m$ to 200,000, the consistency between $tr(\boldsymbol{K}^{4})$ and $L_{4}$ remained when we took $B=50$; but the computational time increased cubically.

“Of note, as $tr\left( \boldsymbol{K}^{4} \right)=\sum_{i=1}^{n} \lambda_{i}^{4}$ is computational expensive when $\boldsymbol{K}$ is large so that only limited sample size and SNP numbers were tested in these 135 simulations; however, the principal results should be retained for an even larger sample size, as well as $\boldsymbol{K}$, but with more expensive computational cost in solving eigenvalues. Biobank-scale performance of the proposed method will be illustrated in UK Biobank 81 traits in **Result 4**.” (**Lines 309-313**)

As practised in this field, the real computational test is UK biobank, and we fully demonstrated the computational capability of our proposed method. Given 10 threads, its computational time was on average 453 mins for the tested UKB traits and the average iteration B=90 given $\eta=0.05$ (Eq 14).

**Further, authors simulated that all SNPs were considered causal after a typical polygenic model. This is not enough information. Exact distributions used to sample SNP effects should be provided. Were the 10k SNPs included in the 50k and 100k sets? If yes, did they have the same effects? Did all SNP had an effect even small or the effect of some SNPs was set to zero? Values of heritability were set to 0, 0.1, and 0.25. Is there any reason that medium to high heritability was not tested? I would strongly recommend to test model performance also with h2 of 0.5, 0.75 and 1 (or close to 1).**

**Reply:** Sorry for confusion. As the purpose of the study is to demonstrate the computational performance of the method, we only adapted a very simple genetic architecture—here polygenic traits for which each marker is causal and sampled from standard normal distribution.

As suggested, we tested the performance of the method by increasing $h^{2}$ to 0.5 (red points), 0.75 (blue points), and 0.9 (green points), respectively. The results were as shown as below. Each cluster had 45 points, which were generated from the combination of LD (0, 0.2, 0.4, 0.6, 0.8), sample size (1,000, 5,000, 10,000), and SNP number (10,000, 50,000, 100,000). As could see in the figure below, the estimated heritability was consistent to the true heritability.

**Letter Figure 1**

**As shown in result 3 “**In addition, we also tested the estimation by setting $h^{2}=0.5$, 0.75, and 0.9, respectively, and the results, as promised by our theory, were consistent to what observed” (lines 352-354)

**For your inquiry “Regarding the case of distributed data, how the model performs in the case of unbalanced data across institutes?”**

Reply: It does not matter. We split the data slightly different but identical results. Please see the figure below, where we simulated 2,000 and 8,000 individuals in each cohort, whereas the split was 4,000 vs 6,000 in our initial submission.

Letter Figure 2

“We also split the data into $n_{1}=$ 2,000 and $n_{2}=$ 8,000 individuals, and, as expected, the results were nearly identical and unbiased (data not shown).” (Lines 372-373).

There is **S1 Data** that implements the above algorithm in R.

**2 Some extra information that is missing and is of interest is the capacity of the computer used to run the analysis, the time required to run each analysis and a comparison with at least two independent and well-known software, as a base-line, that can analyse biobank-scale genomic data. Moreover, as mentioned above, although a github repository is mentioned for the software, no information is provided on the software. It is not clear finally how many iterations are needed to run biobank-scale analysis. In the real data analysis, could you explain the reason to use only unrelated individuals?**

Reply: It is self-supervised by the program to determine B, and Eq 14 is the most important one to determine the stopping rule. See **Figure 5A** panel for various results under different choice of B.

“We also compared the computational efficiency of RHE-reg with GCTA (Yang *et al.*, 2011) and BOLT-REML (Loh *et al.*, 2015) in estimating the heritability on BMI. The comparison was conducted on a sub-dataset in UKB with randomly selected 10,000 individuals and 523,945 SNP markers after filtration. The results indicate significant efficiency improvement in estimating the heritability of complex traits in biobank-scale datasets for RHE-reg, with computation times reduced by 96.6% and 83.8% compared to GCTA and BOLT-REML, respectively (**S2 Table**). More benchmark comparison of the computational performance could be found in earlier studies (Zhou, 2017; Wu and Sankararaman, 2018). Even using a complete dataset, RHE-reg could also complete heritability estimation within an acceptable time (**S3 Table**). In our tested 81 UKB traits, with 10 threads, it on average took 453 mins to finish the analysis of a trait and the average iteration of $B=90$.” (Lines 417-425)

In **S3 Table**, we provided computational time for UKB 81 traits. The average B=90, and costed 453 mins (6.5 hrs) to estimate the heritability of a traits given $\eta=0.05$ and 10 CPUs.

For your inquiry “**In the real data analysis, could you explain the reason to use only unrelated individuals?**” Please refer to our reply to your Question 1. It is kind of a paradigm shift since 2006 that the heritability estimation was completely changed from family-based study (linkage) to population-based studies (association).

**3 The Discussion is too short. All Tables and Figures need to be self-explanatory. Regarding the analysis with the UK-Biobank data (ExData2) there are some discrepancies that need to be discussed. Overall, the standard errors of the h2 estimates are higher compared to those reported in Xu et al. For the trait “Age of primiparous women at birth of child” no estimates were provided. In some cases, there are considerable differences in h2 estimates between Xu et al and the current study, e.g., 0.42 vs 0.17 for the trait “Trunk predicted mass” and 0.73 vs 0.50 for the trait “Trunk predicted mass”. Could you provide with explanations?**

Reply: For tables and figures, we added more notes and figure legends, and we wish they explain themselves better now. For Table 1, we added its detailed calculation into Supplementary Notes. We also updated Figure 2 for a much clearer illustration.

**For your inquiry “Age of primiparous women at birth of child” no estimates were provided.**

The h2 was successfully estimated if no covariate included, but failed if the first 2 PCs and sex included. The RHE algorithm achieves efficient estimation of heritability while ensuring accuracy, but inevitably, there is a certain inflation in the SE of RHE estimation, which may lead to subtle deviations between the estimated heritability and previous research results. In addition, Xu et al.'s research used the first two PCs as covariates for estimating heritability, while in the current study, RHE used the first two PCs plus gender as covariates. We believe this is the main reason for the observed discrepancy in the heritability estimation results of the two studies.

Xu’s work (we coauthored) took much approximation to get SE, because of subsampling technique used rather than the whole data.

“Some inconsistency between GRM-based estimation and randomization estimation, such as the overall correlation of 0.77 for estimated heritability between Xu et al.’s results and the current result, may arise from the different covariates chosen (Xu *et al.*, 2021). In Xu et al.’s work, the heritability was estimated under the first two PCs corrected, while the current randomization method further took gender as extra covariate, this may cause the observed discrepancy especially in the gender-related traits.” (Lines 484-489)

**Overall, I support the publication of this manuscript, but only after addressing all my comments and/or suggestions point by point. Thus, my conclusion is major revision.**

Reply: Thanks for your appreciation. It is really courtesy.

**Minor comments**

In all equations double check the correct use of “~” and “^” for the predicted and estimated values.

**1 L 44 “much faster than REML” – be more precise**

Reply: REML takes $\mathcal{O}\left( n^{2}m \right)$ for making GRM, and $\mathcal{O(}n^{3})$ for inversion. On of the obstacle is that REML could not tackle biobank-scale data anymore when constructing the genetic relationship matrix $\boldsymbol{K}=\frac{1}{m}\boldsymbol{X}\boldsymbol{X}^{T}$, and as an update randomized Haseman-Elston regression is proposed to carry on the biobank-scale data.

“Conventional methods, such as REML, for estimating heritability, like the linear mixed model, often takes computational cost of $\mathcal{O(}n^{2}m+n^{3})$, where $n$ is the sample size and $m$ is the number of markers.” (Lines 53-54)

**2 L 46 “recently a randomized” – I think that a work back in 2018 cannot be considered a s a recent work.**

Reply: Deleted “recently”. However, as it is a very difficult field in dealing biobank-scale data, not much progress has even been made since 2018.

**3 L 63 “horizontal federated learning” – could you elaborate more?**

Reply: It descripts how data is split in distributed computing. An easy example will be

$\frac{3+2}{2+8}=0.5$,site A has 3 and 2, and site B has 2 and 8, it leads to horizontal federated learning. If site A has 5, and site B has 10, it is called vertical federated learning. So, if we take GWAS data as an example, which has SNP data and eHealth data. Horizontal data indicates each institutes has its own set of “SNP+eHealth” and each set of data is generically homogeneous; vertical data indicates that some institute only has SNP data only, and some institutes have eHealth data.

See section “Extended utilities for distributed GWAS datasets”. (Line 226)

**4 L 68 “a large B” – be more precise**

Reply: Actually, the whole paper is about how precisely control $B$. We have revised the text to

“It is clear that a large $B$, indicating more iteration of the presented algorithm, is helpful in improving precision, but it is unsolved how to get an estimate for $B$ and its role in determining the boundaries of key statistics, upon the standard errors of the randomized estimator (Tang *et al.*, 2022).” (Lines 82-84)

**5 L 69 “boundaries of key statistics” – could you explain more?**

Reply: See the reply to **Question 4 (last question)**.

**6 L 95 “of the square of each element” – replace with “of the square of each diagonal element**

Reply: Sorry for any confusion if it is due to our unclearness. See a tiny example, $\boldsymbol{K}\times\boldsymbol{K}^{T}=\left[ \begin{matrix} a & b \\ c & d \end{matrix} \right]\left[ \begin{matrix} a & c \\ b & d \end{matrix} \right]=\left[ \begin{matrix} a^{2}+b^{2} & ac+bd \\ ac+bd & c^{2}+d^{2} \end{matrix} \right]$, and $tr\left( K\times K^{T} \right)=a^{2}+b^{2}+c^{2}+d^{2}=\sum_{i,j}^{2} K_{i,j}^{2}$. So, $tr\left( \boldsymbol{K}^{2} \right)=\sum_{i,j}^{n} K_{i,j}^{2}$ for sure.

**7 L95 “We proved that” – citation is missing**

Reply: In the section of “Estimation for the effective number of markers ($\boldsymbol{m}_{\boldsymbol{e}}$)” we give a brief note on how this proof was made, and more technical details could be found in Zhang et al 2025.

Citation: Precise estimation of in-depth relatedness in biobank-scale datasets using deepKin, *Cell Reports Methods*, 2025, 5:101053

**8 L 97 “correlations” – you mean Pearson correlations?**

Reply: Yes. Squared Pearson’s correlation for LD here.

“We proved that the expectation of $tr\left( \boldsymbol{K}^{2} \right)=\frac{n\left( n+1 \right)}{m_{e}}+n$, where $m_{e}$ is the effective number of markers that depicts the average squared Pearson’s correlations among all genomic markers as often used for measuring linkage disequilibrium (Zhang *et al.*, 2024, 2025; Huang *et al.*, 2023); a brief sketch of how $tr\left( \boldsymbol{K}^{2} \right)$ can be transferred into $m_{e}$ is also presented in the section “Estimation for the effective number of markers” below.” (Lines 112-115)

**9 L 100 tr(Kc) could you provide with the space of the values for c?**

Reply: Here $c$ indicates exponential function, and it takes the value of $1, 2, 3,$ and 4 in this study. In particular, $tr(\boldsymbol{K}^{2})$ and $tr(\boldsymbol{K}^{4})$ are most important two values throughout this study.

“We obtain the estimate of $tr(\boldsymbol{K}^{c})$ according to the properties of matrix algebra, and $c$ is the exponential index and $c$ takes the value of 1, 2, 3, or 4 upon the application in this study.” (Lines 121-122)

**10 Equation 3 – denote “L”**

Reply: L is a notation by itself.

“Where $L_{\mathcal{c,}B}$ is a linear estimator for $tr(\boldsymbol{K}^{c})$, $\boldsymbol{z}_{b}$ is a vector of length $n$ and each element of $\boldsymbol{z}_{b}$ is sampled from the standard normal distribution, and $B$ is the round of iterations.” (Lines 123-124)

**11 L 101 “of of L2,B” remove second “of”**

Reply: Removed. Thanks for catching up.

**12 L 108 “random mating or little inbreeding“ – up to which degree of inbreeding? Be more precise**

Reply: We meant random mating, and “little inbreeding” implicates certain perturbation even under random mating. We deleted “little inbreeding” from the text. (Line 33)

**13 L 114, what is a and b?**

Reply: Here, a and b are two variables. See Appendix I (page 818) in Lynch & Walsh.

$E\left( a \right)=\mu_{a}$ and $var\left( a \right)=\sigma_{a}^{2}$, and similar for b.

$$\left\{ \begin{aligned} a\left\{ \begin{matrix} \mu_{a}=E\left( y^{T}Ky-n \right)=\left[ tr\left( \boldsymbol{K}^{2} \right)-n \right]h^{2} \\ \sigma_{a}^{2}=var\left( \boldsymbol{y}^{T}\left( \boldsymbol{K}-I \right)\boldsymbol{y} \right)=2tr[\Sigma(K-I)\Sigma(K-I)] \end{matrix} \right. \\ b\left\{ \begin{matrix} \mu_{b}=L_{2,B}-n=tr\left( K^{2} \right)-n=\frac{n(n+1)}{m_{e}} \\ \sigma_{b}^{2}=\frac{2}{B}tr(K^{4}) \end{matrix} \right. \end{aligned} \right.$$

The randomized estimator of $h^{2}$ can be seen as a ratio of $\frac{a}{b}$, in which both $a$ and $b$ are variables ($E\left( a \right)=\mu_{a}$ and $var\left( a \right)=\sigma_{a}^{2}$, and analogously for $b$), and, according to Delta method, its sampling variance can be expressed as $var\left( \frac{a}{b} \right)=\frac{1}{\mu_{b}^{2}}\sigma_{a}^{2}-2\frac{\mu_{a}}{\mu_{b}^{3}}cov\left( a,b \right)+\frac{\mu_{a}^{2}}{\mu_{b}^{4}}\sigma_{b}^{2}$, in which the covariance term can be zeroed out in this scenario (Lynch and Walsh, 1998). (Lines 139-143)

**14 L 115, explain μ in the equation**

Reply: We have already addressed it in the above question.

**15 L 118 replace “Tylor” with “Taylor”**

Reply: Corrected.

**16 L 126 “sufficient iterations” – how many?**

Reply: The whole paper is about to determine how many. We here introduce $\eta$ as an indicator, see Eq 14.

$$B=\frac{1}{\eta}\frac{tr\left( \boldsymbol{K}^{4} \right)h^{4}}{\Lambda_{1}}$$

$\eta$ is the precision, which is set 0.05 or 0.1 in this study. As show in our new data (**S3 Table**), the average $B=90$ for 81 tested UKB traits given $\eta=0.05$.

**17 L 127 “large enough” – do you mean going to infinity?**

Reply: According to our study, $B$ is often less than 100. See Fig5 A1 for UKB examples, and our reply to your last question.

**18 L 152 in the equation explain i and j.**

Reply: Here, $i$ and $j$ are subscripts for individuals. See our updated text below

$$E\left[ tr\left( \boldsymbol{K}^{2} \right) \right]=E\left\{ \frac{1}{m^{2}}\sum_{i,j}^{n} \left[ \sum_{k}^{m} \left( x_{ik}x_{jk} \right) \right]^{2} \right\}=E\left\{ \frac{1}{m^{2}}\sum_{i,j}^{n} \left\{ \left[ \sum_{k}^{m} \left( x_{ik}x_{jk} \right) \right]\left[ \sum_{l}^{m} \left( x_{il}x_{jl} \right) \right] \right\} \right\}$$

(Line 189)

**19 L 202 “c institutes” – consider to change the letter “c” in order not to be confused with “c” used in previous equations**

Reply: We changed *c* to *s*. (Line 230)

**20 L 204-205 “yν” and “Xν”, explain subscript “ν”**

Reply: Here, $v$ is the subscript for $\boldsymbol{y}^{T}=[\boldsymbol{y}_{1}^{T}\vdots\boldsymbol{y}_{2}^{T}\vdots\cdots\vdots\boldsymbol{y}_{s}^{T}]$.

For example $\boldsymbol{y}^{T}=[1,2,3,4,5]$ and $\boldsymbol{X}=\left[ \begin{matrix} \begin{matrix} AA & BB \end{matrix} & \begin{matrix} Cc & DD & EE \end{matrix} \\ \begin{matrix} Aa & Bb \end{matrix} & \begin{matrix} cc & Dd & Ee \end{matrix} \end{matrix} \right]$. For $s=2$, $\boldsymbol{y}_{1}^{T}=[1,2]$ and $\boldsymbol{y}_{2}^{T}=[3,4,5]$, and $\boldsymbol{X}_{1}=\left[ \begin{matrix} AA & BB \\ Aa & Bb \end{matrix} \right]$, $\boldsymbol{X}_{2}=\left[ \begin{matrix} Cc & \begin{matrix} DD & EE \end{matrix} \\ cc & \begin{matrix} Dd & Ee \end{matrix} \end{matrix} \right]$.

**21 L 206 explain “F”**

Reply: $F$ is for Frobenius norm of a matrix, $\left\| \boldsymbol{A} \right\|_{F}=\sqrt{tr(AA^{T})}=\sqrt{\sum_{i,j}^{n} A_{ij}^{2}}$. (Lines 255-256)

**22 L 217 “Furthermore, if we have c covariates, and the covariate matrix W is of n x c dimensions” – double check this sentence**

Reply: Thanks for catching up. We have changed $c$ to $w$ as $c$ has been occupied already. Furthermore, we have $w$ covariates, and the covariate matrix W is of $n\times w$ dimensions. (**Supplementary Notes IV**)

**23 L 258 “because n= 1,000 was too small a sample size here” – what does this mean? Were there any convergence issues?**

Reply: Sampling variance for iteration will be too big given $n=1000$, so it was not employed to demonstrate the results. Although it was too small, we felt necessary to mention that.

“We only illustrated the result for $n=5,000$ and 10,000, respectively, because $n=1,000$ was too small a sample size here for efficient convergence (data not shown). LD did not play an important role in determining MSE for the simulated scenarios.” **(Liness 332-334)**

**24 L 265 “much higher h2 took a much greater** $\boldsymbol{B}$**” – please be more precise**

Reply: As $h^{2}$ will be a part of iteration, see Eq 14.

**25 Figure 2 -consider to use same y-axis scale for fair comparisons**

Reply: We have completely rewritten the Result2 and Figure 2. (Lines 320-334)

**26 Figure 3 – explain the blue and red lines. Consider to change h2(B20/B50) to h2(B20) – h2(B50) or h2(B20) / h2(B50) etc. Why negative h2 values are reported?**

Reply: it is due to sampling variance. Under the null hypothesis of zero $\sigma_{h^{2}}^{2}=\frac{2m_{e}}{n^{2}}$, so the estimated heritability definitely falls down below zero. Of course, people are used to report zero even its estimate is below zero.

**27 Figure 4 – what are the negative h2 values?**
Reply: Sampling variance. Similar reason to the question above. See our reply to Q 29 below.

**28 L 337 “a high degree” – consider to change to “a relative high degree”, since the Pearson correlation reported is 0f 0.77**

Reply: Changed. (Line 412)

**29 Figure 5 – what is the meaning of h2 estimates > 1?**

Reply: Sampling variance. Under the null hypothesis of no heritability $\sigma_{h^{2}}^{2}=\frac{2m_{e}}{n^{2}}$, and similar for high heritability, its estimate could possibly be greater than 1.

**30 L 348 and 349 should 30 be replaced with 50?**

Reply: Thanks. We have replaced 30 with 50 now in **Figure 5**.

**31 Figure 6 - what is the meaning of h2 estimates > 1?**

Reply: Two possible reasons. It is because of ill genetic architecture, which is not polygenic anymore; or it is due to sampling variance.

**32 L 385 – 391 are coming “****out of the blue” in the discussion. Consider to make a subheading.**

Reply: Good idea. Yes. it’s about dominance and we have move this paragraph into the end of the paper (Lines 503-509).

Thank you Dr Christos Dadousis again for your critical reading. Wish our revision can improve the overall quality and address your concerns.

**Reviewer #2**

**In this manuscript, the authors developed an analytical solution for scalable estimator of SNP heritability. They conduct simulations and real data application to illustrate the accuracy of their methods. The manuscript is well structured. I have a few questions and suggestions for the authors, which I listed below.**

Reply: Thanks so much for your appreciation and comments, which we will address below!

**Major:**

**1. Equations and Notation. I have several questions regarding the notation used throughout the manuscript. Please review the notation carefully to ensure consistency and clarity across the entire text.**

**a. It would be helpful to define all notations at their first occurrence. For instance, the symbol c in Equation (3), the distribution of z_b, and the variables listed in Table 1 (e.g., q, v) are not clearly defined.**
Reply: $c$ is exponential index, and $\boldsymbol{z}_{b}$ is a vector and each element of $\boldsymbol{z}_{b}$ is sampled from the standard normal distribution.

In Table 1, $q=1-p$ the frequency of the alternative allele, and $v$ the subscript indexes the three genotypes of a locus. We put more detailed calculation of Table 1 into **Supplementary Note V**.

**b. Line 100: I believe the term m^c in the denominator of L_{c,b} should be removed, given that K is defined as XX^t / m. This also differs from Equation (10) in Wu and Sankararaman (2018). Please check for similar inconsistencies in Table 2 and elsewhere in the manuscript.**
Reply: Thanks for catching up. We have corrected the writing as suggested. It does not affect our computational results but only a bug in presentation.

$$\left\{ \begin{matrix} L_{\mathcal{c,}B}=\frac{1}{B}\sum_{b}^{B} \boldsymbol{z}_{b}^{T}\boldsymbol{K}^{\mathcal{c}}\boldsymbol{z}_{b} \\ E\left( L_{c,B} \right)=tr(\boldsymbol{K}^{c}) \\ var\left( L_{c,B} \right)=\frac{2tr\left( \boldsymbol{K}^{2c} \right)}{B} \end{matrix} \right.$$

See Eq 3 (Line 122). It is a different expression but the identical core with Wu and Sankararaman.

**c. Line 158: I find the notation in Equation (11) confusing—particularly the use of tilde m_e, which appears to correspond to vertical RHE-reg in Table 2. Also, is the second part of Equation (11) meant to represent the variance of the estimator** ${\hat{\boldsymbol{m}}}_{\boldsymbol{e}}$**? If so, the corresponding entry in Table 2 should be updated accordingly. Please use hats (^) to indicate estimated values throughout the text.**

Reply: We’ve replaced it with $\hat{m}_{e}$ rather than tilde here. We also update Table 2, for which we added more notes.

**d. Line 242: Should the expression be B/2 rather than 1/B?**

Reply: Corrected. $tr\left( \boldsymbol{K}^{4} \right)\boldsymbol{=}\frac{B}{2}var\boldsymbol{(}\boldsymbol{L}_{2,B}\boldsymbol{)}$. Of note, in practice, we estimate $tr\left( \boldsymbol{K}^{4} \right)$ with $L_{4,B}=\frac{1}{B}\sum_{b=1}^{B} \boldsymbol{z}_{b}^{T}K^{4}\boldsymbol{z}_{b}$, which is more numerically better. See Result 1 (Line 300).

**2. It would strengthen the manuscript to include a comparison between your method and existing approaches, such as that of Wu (2018), in your simulations. Comparing metrics like mean squared error (MSE), computation time, and memory usage would be especially informative.**

Reply: We included comparison with GCTA and BOLTLMM in the analysis of UKBiobank.

“We also compared the computational efficiency of RHE-reg with GCTA (Yang *et al.*, 2011) and BOLT-REML (Loh *et al.*, 2015) in estimating the heritability on BMI. The comparison was conducted on a sub-dataset in UKB with randomly selected 10,000 individuals and 523,945 SNP markers after filtration. The results indicate significant efficiency improvement in estimating the heritability of complex traits in biobank-scale datasets for RHE-reg, with computation times reduced by 96.6% and 83.8% compared to GCTA and BOLT-REML, respectively (**S2 Table**). More benchmark comparison of the computational performance could be found in earlier studies (Zhou, 2017; Wu and Sankararaman, 2018). Even using a complete dataset, RHE-reg could also complete heritability estimation within an acceptable time (**S3 Table**). In our tested 81 UKB traits, with 10 threads, it on average took 453 mins to finish the analysis of a trait and the average iteration of $B=90$.” (Lines 417-425)

**3. Figures. The figure captions should be more self-explanatory. Please clarify what the lines and data points represent.**

**a. Figure 2. I was expecting to see a direct comparison of MSE across the simulation scenarios (or with methods from prior work such as Wu's), which would be more informative than only comparing Lambda_1, Lambda_2, and Lambda_3.**

Reply: Actually, there was an error in Wu and Sankararaman’s paper (see our reply to Q3 of Reviewer 3 below), we contracted them as early as 2021 but some delayed response was received till early 2024. During this process, we spent time to fix their method and developed it into the present method. As far as we know, we and Sankararaman are only two groups who are marching towards algorithms for millions of individuals.

“Of note, $var\left( L_{2,B} \right)=\frac{2tr\left( \boldsymbol{K}^{4} \right)}{B}$, which was incorrectly derived as $var\left( L_{2,B} \right)=\frac{2tr\left( \boldsymbol{K}^{2} \right)}{B}$ in Wu and Sankararaman’s work (Wu and Sankararaman, 2018), and to fix their problem directly led to the present work.” (Lines 128-130)

A snapshot from Wu and Sankararam 2018. The highlighted part is incorrect and led to various problems in their paper.

So, there is some uncertainty to compare with their methods. However, we have rewritten Result 2 and hope to deliver the message more clearly. (Line 319 and updated Figure 2; see our reply below).

**b. Line 261-262, The caption states that Figure 2 shows how quickly B can reduce Lambda_2/B, but the y-axis shows only Lambda_2. Please clarify.**

Reply: We have rewritten the paragraph to deliver the message much clearer. (Line 319 and updated Figure 2; see our reply below).

**c. Line 262-266. Please elaborate on how Lambda_1, Lambda_2, and Lambda_3 relate to MSE, and what insights Figure 2 provides. The differing axis scales make it hard to interpret your conclusions.**

Reply:

Result 2: MSE of RHE-reg

“In **Eq 7**, $MSE\left( \hat{h}^{2} \right)=2\left( \frac{m_{e}}{n^{2}} \right)^{2}\left( \Lambda_{1}+\frac{tr(\boldsymbol{K}^{4})\cdot h^{4}}{B} \right)+4\left( \frac{m_{e}}{n^{2}} \right)^{4}{\cdot\left[ \frac{tr\left( \boldsymbol{K}^{4} \right)}{B} \right]}^{2}\cdot h^{4}$, and, for ease of presentation, we reorganize **Eq 7** by denoting with $\Lambda_{0}=\frac{n^{2}}{m_{e}}$, $\Lambda_{2}=tr\left( \boldsymbol{K}^{4} \right){\cdot h}^{4}$, and $\Lambda_{3}=2\left( \frac{m_{e}}{n^{2}} \right)^{2}{\cdot tr\left( \boldsymbol{K}^{4} \right)\cdot h}^{2}$. So $MSE\left( \hat{h}^{2} \right)=\frac{2}{\Lambda_{0}}\left( \Lambda_{1}+\frac{\Lambda_{2}}{B} \right)+\left( \frac{\Lambda_{3}}{B} \right)^{2}$. We compared these three components $\Lambda_{1}$, $\frac{\Lambda_{2}}{B}$, and $\left( \frac{\Lambda_{3}}{B} \right)^{2}$, respectively. The relative ratio between $\Lambda_{1}$ and $\frac{\Lambda_{2}}{B}$ indicates how $B$ can reduce the standard error, and between $\Lambda_{1}$ and $\left( \frac{\Lambda_{3}}{B} \right)^{2}$ indicates how fast the bias will be eliminated by $B$.

The first row of **Figure** **2** reflected how quickly $B$ could reduce $\frac{\Lambda_{2}}{B}$ and $\frac{\Lambda_{3}}{B}$, as indicated by the vertically dashed lines in each panel. As $B$ increases from 10 to 50, $\frac{\Lambda_{2}}{B}$ was shrunk much faster towards zero (top panels). In contrast, $\Lambda_{3}$, which was weighted down by $B^{2}$ (the bottom panels), was seemed a less important term in the MSE of $h^{2}$ via RHE-reg, and vanished much faster than $\Lambda_{2}$ after $B$ iterations. Actually, the scales of $\frac{\Lambda_{2}}{B}$ and $\left( \frac{\Lambda_{3}}{B} \right)^{2}$ were very different, and the latter was much smaller. Furthermore, a much higher $h^{2}$ took a much greater $B$ in comparing the regressions across all scenarios. This is consistent with the mathematical relationship whereby, the terms $\frac{\Lambda_{2}}{B}$ and $\frac{\Lambda_{3}^{2}}{B^{2}}$ decrease with a larger $B$, thereby reducing their contribution to MSE. We only illustrated the result for $n=5,000$ and 10,000, respectively, because $n=1,000$ was too small a sample size here for efficient convergence (data not shown). LD did not play an important role in determining MSE for the simulated scenarios.” (Lines 319-334 and Figure 2).

**Minor**

**1 Line 287. “The fitted regression” — of what? It would be better to plot the data point and provide a more detailed explanation in the caption. Plus, the color legend of B is missing.**

Reply: We have completely rewritten this paragraph.

Result 2: MSE of RHE-reg

“In **Eq 7**, $MSE\left( \hat{h}^{2} \right)=2\left( \frac{m_{e}}{n^{2}} \right)^{2}\left( \Lambda_{1}+\frac{tr(\boldsymbol{K}^{4})\cdot h^{4}}{B} \right)+4\left( \frac{m_{e}}{n^{2}} \right)^{4}{\cdot\left[ \frac{tr\left( \boldsymbol{K}^{4} \right)}{B} \right]}^{2}\cdot h^{4}$, and, for ease of presentation, we reorganize **Eq 7** by denoting with $\Lambda_{0}=\frac{n^{2}}{m_{e}}$, $\Lambda_{2}=tr\left( \boldsymbol{K}^{4} \right){\cdot h}^{4}$, and $\Lambda_{3}=2\left( \frac{m_{e}}{n^{2}} \right)^{2}{\cdot tr\left( \boldsymbol{K}^{4} \right)\cdot h}^{2}$. So $MSE\left( \hat{h}^{2} \right)=\frac{2}{\Lambda_{0}}\left( \Lambda_{1}+\frac{\Lambda_{2}}{B} \right)+\left( \frac{\Lambda_{3}}{B} \right)^{2}$. We compared these three components $\Lambda_{1}$, $\frac{\Lambda_{2}}{B}$, and $\left( \frac{\Lambda_{3}}{B} \right)^{2}$, respectively. The relative ratio between $\Lambda_{1}$ and $\frac{\Lambda_{2}}{B}$ indicates how $B$ can reduce the standard error, and between $\Lambda_{1}$ and $\left( \frac{\Lambda_{3}}{B} \right)^{2}$ indicates how fast the bias will be eliminated by $B$.

The first row of **Figure** **2** reflected how quickly $B$ could reduce $\frac{\Lambda_{2}}{B}$ and $\frac{\Lambda_{3}}{B}$, as indicated by the vertically dashed lines in each panel. As $B$ increases from 10 to 50, $\frac{\Lambda_{2}}{B}$ was shrunk much faster towards zero (top panels). In contrast, $\Lambda_{3}$, which was weighted down by $B^{2}$ (the bottom panels), was seemed a less important term in the MSE of $h^{2}$ via RHE-reg, and vanished much faster than $\Lambda_{2}$ after $B$ iterations. Actually, the scales of $\frac{\Lambda_{2}}{B}$ and $\left( \frac{\Lambda_{3}}{B} \right)^{2}$ were very different, and the latter was much smaller. Furthermore, a much higher $h^{2}$ took a much greater $B$ in comparing the regressions across all scenarios. This is consistent with the mathematical relationship whereby, the terms $\frac{\Lambda_{2}}{B}$ and $\frac{\Lambda_{3}^{2}}{B^{2}}$ decrease with a larger $B$, thereby reducing their contribution to MSE. We only illustrated the result for $n=5,000$ and 10,000, respectively, because $n=1,000$ was too small a sample size here for efficient convergence (data not shown). LD did not play an important role in determining MSE for the simulated scenarios.” (Lines 319-334 and Figure 2).

**2 Figure 4. Including a 45-degree reference line would be helpful. Also, please explain what the data points represent.**

Reply: Thank you. We have added the identity line (y=x) in Figure 4.

Point colors represent the simulated heritability. Each scenario was repeated 10 times. Each data point represents a repeat by generating random SNP effects.

**3 Line 359. Please define what “split 1/2” and “split 2/1” refer to in vertical RHE-reg.**

Reply: Estimate denominator and numerator from data 1 and 2, or data 2 and 1.

Of **Eq 15**, ${\hat{\tilde{h}}}^{2}=\tilde{m}_{e}\cdot\frac{(\boldsymbol{y}^{T}\boldsymbol{Ky}-n)}{n^{2}}$ indicates that $\tilde{m}_{e}$ and $\frac{(\boldsymbol{y}^{T}\boldsymbol{Ky}-n)}{n^{2}}$ can be from two independent sources. Consequently, we split each UKB trait evenly into halves to test the v-RHE-reg, and Eq 15 had four possible combinations: 1) split 1/1: both $\tilde{m}_{e}$ and $\frac{(\boldsymbol{y}^{T}\boldsymbol{Ky}-n)}{n^{2}}$ were estimated from split 1; 2) split 2/1: $\tilde{m}_{e}$ was estimated from split 2 and $\frac{(\boldsymbol{y}^{T}\boldsymbol{Ky}-n)}{n^{2}}$ split 1; 3) split 1/2: $\tilde{m}_{e}$ was estimated from split 1 and $\frac{(\boldsymbol{y}^{T}\boldsymbol{Ky}-n)}{n^{2}}$ split 2; 4) both $\tilde{m}_{e}$ and $\frac{(\boldsymbol{y}^{T}\boldsymbol{Ky}-n)}{n^{2}}$ were estimated from split 2. So, we had four estimators as below

$$\left\{ \begin{matrix} \begin{matrix} h_{1,1}^{2}=\left[ \tilde{m}_{e} \right]_{1}\cdot\left[ \frac{\left( \boldsymbol{y}^{T}\boldsymbol{Ky}-n \right)}{n^{2}} \right]_{1}, \mathrm{split} 1/1 \\ h_{1,2}^{2}=\left[ \tilde{m}_{e} \right]_{1}\cdot\left[ \frac{\left( \boldsymbol{y}^{T}\boldsymbol{Ky}-n \right)}{n^{2}} \right]_{2}, \mathrm{split} 2/1 \end{matrix} \\ \begin{matrix} h_{2,1}^{2}=\left[ \tilde{m}_{e} \right]_{2}\cdot\left[ \frac{\left( \boldsymbol{y}^{T}\boldsymbol{Ky}-n \right)}{n^{2}} \right]_{1}, \mathrm{split} 1/2 \\ h_{2,2}^{2}=\left[ \tilde{m}_{e} \right]_{2}\cdot\left[ \frac{\left( \boldsymbol{y}^{T}\boldsymbol{Ky}-n \right)}{n^{2}} \right]_{2}, \mathrm{split} 2/2 \end{matrix} \end{matrix} \right.$$

Lines 441-446.

Thanks again for your critical reading. Wish our revision can improve the overall quality and address your concerns.

**Reviewer #3**

**Qi at al developed an analytical and computational solution for estimating SNP-heritability at biobank-scale scale data, which is an extremely important problem but often challenged because of computational burden. I very much appreciate the authors’ theoretical effort to attack this important problem and the work is also impressive. My major comments are trying to help the presentation of the manuscript.**

Reply: Thanks for your appreciation!

**I found the current manuscript is not easy to understand. In the main text, there are a lot of mathematical formulas and derivations, but many steps have been missed, which are difficult to follow. I suggest the authors present the final formula with clear notation definitions and leaving detailed mathematical derivations in Supplementary Note. This should improve readability. I will try to give my specific suggestions in my comments. I also suggest the authors carefully check all the mathematical formulas and make sure they are correct.**

**Q1: Line 96-97, it will be good to point out where the prove of E(tr(K2)), and E(h ̂^2) are. It is also confused why E(h ̂^2) is still involved y? It will be good to add more details for the derivations, such as a Supplementary note.**

Reply: We can replace $y$ with correlation or beta, upon the context. $E\left( \hat{h}^{2} \right)=\frac{m_{e}}{n^{2}}\left( \boldsymbol{y}^{T}\boldsymbol{Ky}-n \right)=\frac{\bar{r}_{mq}^{2}}{\bar{r}_{m}^{2}}h^{2}$, which is an established results for a typical polygenic trait. $\bar{r}_{mq}^{2}$ the averaged LD between a marker and a QTL, which is not observed, and $\bar{r}_{m}^{2}$ is averaged LD between any pair of markers. As $\frac{\bar{r}_{mq}^{2}}{\bar{r}_{m}^{2}}\leq1$, $E\left( \hat{h}^{2} \right)=\frac{\bar{r}_{mq}^{2}}{\bar{r}_{m}^{2}}h^{2}\leq h^{2}$.

“Therefore, the expectation for the preliminary estimator of $h^{2}$ is $E\left( \hat{h}^{2} \right)=\frac{m_{e}}{n^{2}}\left( y^{T}Ky-n \right)=\frac{\bar{r}_{\mathrm{mq}}^{2}}{\bar{r}_{m}^{2}}h^{2}$ for a typical polygenic trait as established (Chen, 2014; Zhou, 2017); $\bar{r}_{\mathrm{mq}}^{2}$ is the averaged LD between a marker and a causal variant, and $\bar{r}_{m}^{2}=\frac{1}{m_{e}}=\frac{\sum_{k,l}^{m} \rho_{\mathrm{kl}}^{2}}{m^{2}}$ is the averaged LD between any pair of markers – including the LD of a marker with itself.” (Lines 115-118)

Reference

Guo-Bo Chen, Estimating heritability of complex traits from genome-wide association studies using IBS-based Haseman-Elston regression, ***Front Genet***, 2014, 5:107; this paper gives a method-of-moment framework for estimating heritability for complex traits and provides analytical solution for the estimated heritability. However, the involvement of $\boldsymbol{K}=\frac{1}{m}\boldsymbol{X}\boldsymbol{X}^{T}$ leads to high computational cost and not a good tool for biobank-scale data (from the author himself).

Xiang Zhou, A unified framework for variance component estimation with summary statistics in genome-wide association studies, ***Annals of Applied Statistics***, 2017, 11:2027-51; this paper gives the identity of $tr\left( \boldsymbol{K}^{2} \right)=\frac{n^{2}}{m_{e}}+n$, but it fails to estimate $m_{e}$ in biobank scale datasets.

**Q2: Line 100, In equation (3), it will be better to introduce L_(c,B) first before writing the equation. From my calculation, the current definition of L_(c,B) leads to (E(L_(c,B))=1/m^c tr(K^c), which is inconsistent with the second equation. I think m^c only presents when K is writing as XX(T). I think the current definition of L_(c,B) (the first equation in equations (3)) does not have the term 1/m^c.**

Reply: Similar to the question raised by reviewer 2. I should correct it. See Eq 3

$$\left\{ \begin{matrix} L_{\mathcal{c,}B}=\frac{1}{B}\sum_{b}^{B} \boldsymbol{z}_{b}^{T}\boldsymbol{K}^{\mathcal{c}}\boldsymbol{z}_{b} \\ E\left( L_{c,B} \right)=tr(\boldsymbol{K}^{c}) \\ var\left( L_{c,B} \right)=\frac{2tr\left( \boldsymbol{K}^{2c} \right)}{B} \end{matrix} \right.$$

Lines 122-123.

**Q3: In addition, the equation var(L_(c,B) )=(2tr(K^2c))/B in equations (3) is not the same as the equation in Wu and Sankararaman (2018). I believe the authors’ equation is correct, but I think the authors should point out the inconsistence if this is true.**

Reply: I’m reluctant to say they were incorrect, they were wrong even though.

“Of note, $var\left( L_{2,B} \right)=\frac{2tr\left( \boldsymbol{K}^{4} \right)}{B}$, which was incorrectly derived as $var\left( L_{2,B} \right)=\frac{2tr\left( \boldsymbol{K}^{2} \right)}{B}$ in Wu and Sankararaman’s work (Wu and Sankararaman, 2018), and to fix that problem led to this work.” (Lines 128-130)

**Q4: Again, line 110, L_(2,B) does not have 1/m2.**

Reply: corrected.

**Q5: Equations (5), (6) and (7) need additional details. Again, a supplementary note will be good.**

Reply: We feel it is better to add more details around the original text. Line 139-151. However, we do include a more detailed Supplementary Notes for other relevant materials. See our uploaded Supplementary Notes.

**Q6: In equation (8), what is σ ̃_(h^2) refers? There is no definition.**

Reply: Sampling variance of the estimated heritability, see Eq 5.

“Given the estimation of heritability, we can construct the *z*-score statistic below:

$$z_{1}=\frac{\hat{h}^{2}}{\hat{\sigma}_{h^{2}}}=\left( \frac{n^{2}}{{\sqrt{2}m}_{e}} \right)\frac{\hat{h}^{2}}{\sqrt{\Lambda_{1}}\sqrt{1+\frac{\eta}{B}}}$$

(8)

in which $\eta=\frac{tr\left( \boldsymbol{K}^{4} \right)h^{4}}{\Lambda_{1}}$, a quantity that will be zeroed out after sufficient iterations, and $\hat{\sigma}_{h^{2}}$ can be estimated from **Eq 5**.” (Lines 154-156).

**Q7: Line 129 and equation (10), why need z3? Should z3 be the same as z2? Confused.**

Reply: In summary, given $B$ iterations the test statistic observed is $z_{1}$, which is subject to the realized values of $m_{e}$, $\Lambda_{1}$, and $\hat{h}^{2}$. $z_{2}$ is the expected optimized test statistic when $B$ is very large and zeroed out all uncertainty due to iteration. $z_{3}$ is a reconstruction of $z_{1}$, and $\frac{z_{3}}{z_{1}}=\sqrt{1+\frac{\eta}{B}}$, indicating how a larger $B$ seem to bring out advantage in such as a more significant *p*-value.

**Q8: Line 151, the title is “Estimation for the effective number of markers…”. But it is actually for estimating tr(K2).**

Reply: The transformation between $tr(K^{2})$ and LD and m_e are now documented in our supplementary notes, and in the main text we put the results directly there.

Actually $tr\left( K^{2} \right)=\frac{n^{2}}{m_{e}}+n$ can be found in Zhou Xiang (2017, A unified framework for variance component estimation with summary statistics in genome-wide association studies), and in its supplementary note page 26, you may find something amazing about the transformation between K and ld.

**Q9: Line 152, it should be E(tr(K2)) rather than tr(K2). The derivation should add additional detail, perhaps a Supplementary note.**

Reply: Yes, E(tr(K2)). Correctly. We put its details into Supplementary Notes.

**Q10: Line 153, the fourth term summation should be m rather than n. I guess this term comes from ∑_(i≠j)^n∑_(k≠l)^m[x_(i,k)^2 x_(j,l)^2]. Since i≠j, the expectation of this term should be n(n-1)m(m-1). I am not clear whether this difference will affect the conclusion.**

Reply: Corrected. Thanks. We put its details into Supplementary Notes.

**Q11: Line 176, y ̃_b is not defined.**

Reply: typo, $z_{b}$ actually.

“We define the ratio $\eta$ as below

$$\eta=\frac{\mathrm{tr}\left( K^{4} \right)h^{4}}{\Lambda_{1}}$$

(13)

in which $\mathrm{tr}\left( K^{4} \right)$ can be estimated as $\hat{tr(K^{4})}=L_{4,B}=\frac{1}{B}\sum_{b}^{B} z_{b}^{T}K^{4}z_{b}$ as above.” (Lines 219-220)

**Q12: Line 191, what is the numerator of Eq 5 refers?**

Reply: Should have been **Eq 4**. Sorry for the typo. (Line 236)

**Q13: Line 194, additional detail will be good.**

Reply: See supplementary note for more details.

We copy it from the Supplementary Note III

For vertical RHE-reg

$${\hat{\tilde{h}}}^{2}=\frac{\tilde{n}^{2}(\boldsymbol{y}^{T}\boldsymbol{Ky}-n)}{n^{2}(\tilde{L}_{2,B}-\tilde{n})}$$

If the numerator and denominator are estimated from different datasets, according to Delta method, its sampling variance can be expressed as $var\left( \frac{a}{b} \right)=\frac{1}{\mu_{b}^{2}}\sigma_{a}^{2}-2\frac{\mu_{a}}{\mu_{b}^{3}}cov\left( a,b \right)+\frac{\mu_{a}^{2}}{\mu_{b}^{4}}\sigma_{b}^{2}$, in which the covariance term can be zeroed out in this scenario,

$$\left\{ \begin{aligned} a\left\{ \begin{matrix} \mu_{a}=E\left( y^{T}Ky-n \right)=\left[ tr\left( \boldsymbol{K}^{2} \right)-n \right]h^{2} \\ \sigma_{a}^{2}=var\left( \boldsymbol{y}^{T}\left( \boldsymbol{K}-I \right)\boldsymbol{y} \right)=2tr[\Sigma(K-I)\Sigma(K-I)] \end{matrix} \right. \\ b\left\{ \begin{matrix} \mu_{b}=\tilde{L}_{2,B}-\tilde{n}=tr\left( \tilde{K}^{2} \right)-\tilde{n}=\frac{\tilde{n}(\tilde{n}+1)}{\tilde{m}_{e}} \\ \sigma_{b}^{2}=\frac{2}{B}tr(\tilde{K}^{4}) \end{matrix} \right. \end{aligned} \right.$$

$$var\left( \frac{a}{b} \right)=\frac{1}{\mu_{b}^{2}}\sigma_{a}^{2}+\frac{\mu_{a}^{2}}{\mu_{b}^{4}}\sigma_{b}^{2}=\frac{1}{tr\left( \tilde{K}^{2} \right)-\tilde{n}}2tr\left[ \Sigma\left( K-I \right)\Sigma\left( K-I \right) \right]+\frac{\left\{ \left[ tr\left( \boldsymbol{K}^{2} \right)-n \right]h^{2} \right\}^{2}}{\left[ tr\left( \tilde{K}^{2} \right)-\tilde{n} \right]^{4}}\frac{2}{B}tr\left( \tilde{K}^{4} \right)=\frac{1}{\left[ tr\left( \tilde{K}^{2} \right)-\tilde{n} \right]^{2}}[2tr\left[ \Sigma\left( K-I \right)\Sigma\left( K-I \right) \right]+\left[ \frac{tr\left( \boldsymbol{K}^{2} \right)-n}{tr\left( {\tilde{\boldsymbol{K}}}^{2} \right)-\tilde{n}} \right]^{2}h^{4}\frac{2}{B}tr\left( \tilde{K}^{4} \right)]$$

As $E\left[ tr\left( K^{2} \right) \right]=\frac{n^{2}}{m_{e}}+n$, and $E\left[ tr\left( \tilde{K}^{2} \right) \right]=\frac{\tilde{n}^{2}}{\tilde{m}_{e}}+\tilde{n}$. So $\left[ \frac{tr\left( \boldsymbol{K}^{2} \right)-n}{tr\left( {\tilde{\boldsymbol{K}}}^{2} \right)-\tilde{n}} \right]^{2}=\left( \frac{n^{2}}{\tilde{n}^{2}}\frac{\tilde{m}_{e}}{m_{e}} \right)^{2}$

$$var\left( \frac{a}{b} \right)=\left( \frac{\tilde{m}_{e}}{\tilde{n}^{2}} \right)^{2}\left[ \Lambda_{1}+\left( \frac{n^{2}}{\tilde{n}^{2}}\frac{\tilde{m}_{e}}{m_{e}} \right)^{2}h^{4}\frac{2}{B}tr\left( \tilde{K}^{4} \right) \right]$$

If the reference panel is of similar sample size to

$$var\left( \frac{a}{b} \right)\approx\left( \frac{\tilde{m}_{e}}{\tilde{n}^{2}} \right)^{2}\left[ \Lambda_{1}+\left( \frac{\tilde{m}_{e}}{m_{e}} \right)^{2}h^{4}\frac{2}{B}tr\left( \tilde{K}^{4} \right) \right]$$

If furthermore, the numerator and denominator are from the same ancestry

$$var\left( \frac{a}{b} \right)\approx\left( \frac{\tilde{m}_{e}}{\tilde{n}^{2}} \right)^{2}\left[ \Lambda_{1}+h^{4}\frac{2}{B}tr\left( \tilde{K}^{4} \right) \right]$$

In the main text, we only consider $n\approx\tilde{n}$ for simplicity and was the cases considered in **Fig 6** that UKB was evenly split to two parts.

**Q14: Line 233-234, How LD was simulated? Was the LD randomly sample from 0, 0.2, …, 0.8?**

Reply: LD were simulated for five levels. For each simulated data, LD two consecutive markers was universally one of LD the values. The detailed simulation protocol can be found in Zhang et al 2025 (*Cell Reports Methods*).

**Q15: Line 163-264, it states “Λ3 was seemed a less important… and vanished much faster than Λ2 “. Why it did vanish?**

Reply: We have rewritten this paragraph. (Lines 319-334). After iteration $\left( \frac{\Lambda_{3}}{B} \right)^{2}=4\left( \frac{m_{e}}{n^{2}} \right)^{4}{\cdot\left[ \frac{tr\left( \boldsymbol{K}^{4} \right)}{B} \right]}^{2}\cdot h^{4}$ will vanish because of large B, and similar for $\frac{\Lambda_{2}}{B}=\frac{tr(\boldsymbol{K}^{4})\cdot h^{4}}{B}$.

**Result 2: MSE of RHE-reg**

“In **Eq 7**, $MSE\left( \hat{h}^{2} \right)=2\left( \frac{m_{e}}{n^{2}} \right)^{2}\left( \Lambda_{1}+\frac{tr(\boldsymbol{K}^{4})\cdot h^{4}}{B} \right)+4\left( \frac{m_{e}}{n^{2}} \right)^{4}{\cdot\left[ \frac{tr\left( \boldsymbol{K}^{4} \right)}{B} \right]}^{2}\cdot h^{4}$, and, for ease of presentation, we reorganize **Eq 7** by denoting with $\Lambda_{0}=\frac{n^{2}}{m_{e}}$, $\Lambda_{2}=tr\left( \boldsymbol{K}^{4} \right){\cdot h}^{4}$, and $\Lambda_{3}=2\left( \frac{m_{e}}{n^{2}} \right)^{2}{\cdot tr\left( \boldsymbol{K}^{4} \right)\cdot h}^{2}$. So $MSE\left( \hat{h}^{2} \right)=\frac{2}{\Lambda_{0}}\left( \Lambda_{1}+\frac{\Lambda_{2}}{B} \right)+\left( \frac{\Lambda_{3}}{B} \right)^{2}$. We compared these three components $\Lambda_{1}$, $\frac{\Lambda_{2}}{B}$, and $\left( \frac{\Lambda_{3}}{B} \right)^{2}$, respectively. The relative ratio between $\Lambda_{1}$ and $\frac{\Lambda_{2}}{B}$ indicates how $B$ can reduce the standard error, and between $\Lambda_{1}$ and $\left( \frac{\Lambda_{3}}{B} \right)^{2}$ indicates how fast the bias will be eliminated by $B$.

The first row of **Figure** **2** reflected how quickly $B$ could reduce $\frac{\Lambda_{2}}{B}$ and $\frac{\Lambda_{3}}{B}$, as indicated by the vertically dashed lines in each panel. As $B$ increases from 10 to 50, $\frac{\Lambda_{2}}{B}$ was shrunk much faster towards zero (top panels). In contrast, $\Lambda_{3}$, which was weighted down by $B^{2}$ (the bottom panels), was seemed a less important term in the MSE of $h^{2}$ via RHE-reg, and vanished much faster than $\Lambda_{2}$ after $B$ iterations. Actually, the scales of $\frac{\Lambda_{2}}{B}$ and $\left( \frac{\Lambda_{3}}{B} \right)^{2}$ were very different, and the latter was much smaller. Furthermore, a much higher $h^{2}$ took a much greater $B$ in comparing the regressions across all scenarios. This is consistent with the mathematical relationship whereby, the terms $\frac{\Lambda_{2}}{B}$ and $\frac{\Lambda_{3}^{2}}{B^{2}}$ decrease with a larger $B$, thereby reducing their contribution to MSE. We only illustrated the result for $n=5,000$ and 10,000, respectively, because $n=1,000$ was too small a sample size here for efficient convergence (data not shown). LD did not play an important role in determining MSE for the simulated scenarios.” (Lines 319-334)

**Q16: Figure 2 includes many symbols which cannot be recognized. It is possible due to the format.**

Reply: We have updated Figure 2 for a possible much clearer presentation.

**Q17: Line 273, it states “the sample size Λ0=…”. Why does it refer to sample size?**

Reply: It’s an established result that $\sigma_{h^{2}}^{2}=\frac{2m_{e}}{n^{2}}$ under REML, see Eq 4 in Visscher et al PLoS Genetics 2014, 10:e1004296 (Statistical Power to Detect Genetic (Co)Variance of Complex Traits Using SNP Data in Unrelated Samples).

“$\frac{2m_{e}}{n^{2}}$ is the sampling variance of REML when $h^{2}=0$ (Visscher *et al.*, 2014).” (Line 346).

**Q18: Figure 3 only plotted the regression lines. Should be all the points be added so the readers can see how many data points were used in the regressions?**

Reply: Ok, we added more legends at the end of Figure 3. There are 45 points in each colour in each plot.

**Q19: Line 336-338, it states the Pearson’s correlation coefficient 0.77. Should this correlation suggest the discrepancy between the current estimates and that by Xu at all?**

Reply: The RHE algorithm achieves efficient estimation of heritability while ensuring accuracy, but inevitably, there is a certain inflation in the SE of RHE estimation, which may lead to subtle deviations between the estimated heritability and previous research results. In addition, Xu et al.'s research used the first two PCs as covariates for estimating heritability, while in the current study, RHE used the first two PCs plus gender as covariates. We believe this is the main reason for the observed discrepancy in the heritability estimation results of the two studies.

“Some inconsistency between GRM-based estimation and randomization estimation, such as the overall correlation of 0.77 for estimated heritability between Xu et al.’s results and the current result, may arise from the different covariates chosen (Xu *et al.*, 2021). In Xu et al.’s work, the heritability was estimated under the first two PCs corrected, while the current randomization method further took gender as extra covariate, this may cause the observed discrepancy especially in the gender-related traits.” (Lines 484-489)

**Q20: I think the authors should add computational time for different B to see the improvement.**

Reply: Thanks for your comments. We added the computational time for all 81 continuous traits in this revision. Please find the results in **S3 Table**.

“We also compared the computational efficiency of RHE-reg with GCTA (Yang *et al.*, 2011) and BOLT-REML (Loh *et al.*, 2015) in estimating the heritability on BMI. The comparison was conducted on a sub-dataset in UKB with randomly selected 10,000 individuals and 523,945 SNP markers after filtration. The results indicate significant efficiency improvement in estimating the heritability of complex traits in biobank-scale datasets for RHE-reg, with computation times reduced by 96.6% and 83.8% compared to GCTA and BOLT-REML, respectively (**S2 Table**). More benchmark comparison of the computational performance could be found in earlier studies (Zhou, 2017; Wu and Sankararaman, 2018). Even using a complete dataset, RHE-reg could also complete heritability estimation within an acceptable time (**S3 Table**). In our tested 81 UKB traits, with 10 threads, it on average took 453 mins to finish the analysis of a trait and the average iteration of $B=90$.” (Lines 417-425)
