# Supplementary material for: Analytical and computational solution for the estimation of SNP-heritability in biobank-scale and distributed datasets
Source: PLoS Comput Biol. 2025 Oct 21;21(10):e1013568. doi: 10.1371/journal.pcbi.1013568 (PMC12539748; doi:10.1371/journal.pcbi.1013568)
Supplement: Attachment — Submitted filename: LetterRebuttle.docx [file pcbi.1013568.s008.docx]

Reviewer #1:

**Dear authors,**

**I would like to thank you very much for your work and apologise for any delays in the reviewing process.**

**There are few grammar errors that I believe will be corrected during final checks from PLOS computational biology.**

**At line 235 replace c institutes with s institutes.**

>> Reply: Fixed. Thanks. Line 249.

Thanks again for all your constructive comments!

Reviewer #2:

**Thank you for your efforts in revising the manuscript. My previous concerns have been greatly addressed. I just have one remaining question regarding new Figure 2.**

**Could you clarify why the scale of λ₂/B in the current Figure 2 reaches into the hundreds or thousands, while λ₂ was previously shown to be in the range of hundredths or lower (similar concern with λ3)? Also, the color coding appears off. In the fourth panel, the green dashed is supposed to indicate the mean along the x-axis, but there is no green dots on its right side. Panel 1 has the same issue.**

**As my previously comment, it would be better to draw the MSE value across the simulation scenarios.**

>> Reply: We reorganized Fig 2, which reflects how MSE changes with B (top row), and how iteration eliminate sampling variance (bottom row). Lines 320-337.

**And it would be better to add a color legend in Figure 3.**

>> Reply: We checked Figure 3, which has its colour legend complete.

Thanks again for all your constructive comments!
